# Essential proteins and possible therapeutic targets of *Wolbachia* endosymbiont and development of FiloBase-a comprehensive drug target database for Lymphatic filariasis

**DOI:** 10.1038/srep19842

**Published:** 2016-01-25

**Authors:** Om Prakash Sharma, Muthuvel Suresh Kumar

**Affiliations:** 1 Centre for Bioinformatics, School of Life Science, Pondicherry University, Pondicherry-605014, India

## Abstract

Lymphatic filariasis (Lf) is one of the oldest and most debilitating tropical diseases. Millions of people are suffering from this prevalent disease. It is estimated to infect over 120 million people in at least 80 nations of the world through the tropical and subtropical regions. More than one billion people are in danger of getting affected with this life-threatening disease. Several studies were suggested its emerging limitations and resistance towards the available drugs and therapeutic targets for Lf. Therefore, better medicine and drug targets are in demand. We took an initiative to identify the essential proteins of *Wolbachia* endosymbiont of *Brugia malayi*, which are indispensable for their survival and non-homologous to human host proteins. In this current study, we have used proteome subtractive approach to screen the possible therapeutic targets for *wBm*. In addition, numerous literatures were mined in the hunt for potential drug targets, drugs, epitopes, crystal structures, and expressed sequence tag (EST) sequences for filarial causing nematodes. Data obtained from our study were presented in a user friendly database named FiloBase. We hope that information stored in this database may be used for further research and drug development process against filariasis. URL: http://filobase.bicpu.edu.in.

Lymphatic filariasis (Lf) is one of the most neglected tropical diseases in many countries. It causes major public health problem^1^ such as physical disability, disfiguring and chronic morbidity^2^. It is predominantly caused by *Wuchereria bancrofti* (*W. bancrofti*) and *Brugia malayi* (*B. malayi*) and being transmitted in the host body through the bite of *Anopheles* mosquitoes (*An farauti, An punctulatus, An koliensis* and others) and *Culex quinquefasciatus*^3^.

Since 20 years, three classical drugs named Diethylcarbamazine (DEC), Ivermectin (IVM) and Albendazole (ALB)^4^ are in practice to cure this disease. It is noticeable that currently these drugs are being used in the Global Programme to Eliminate Lymphatic Filariasis (GPELF) program, which has targeted to eliminate this disease by 2020^5^. Several studies have shown that Mass Drug Administration (MDA) does not cure filarial infections completely; it only reduces new infections by clearing larvae from the human blood^2^,^6^,^7^,^8^. DEC and IVM kill microfilaria (mf) but it remains ineffective against adult worms^4^,^9^. Moreover, in African countries, DEC causes severe side-effects in the Onchocerca infected patient and IVM cannot be prescribed to the Loa-loa patient^10^,^11^,^12^. Thus, hunting of widespread macrofilaricides through the most essential proteins of pathogens are still remains a big challenge to wipe out this disease^13^. To overcome with this limited drug and drug targets for Lf, we initiated our research project to recognize the novel therapeutic targets for Lf, which should be essential for their survival and does not show any similarity with the human host proteins.

In the past few years, several investigators have made extensive efforts to identify better drugs and therapeutic targets to enhance the treatment of Lf^14^,^15^,^16^,^17^,^18^,^19^,^20^,^21^,^22^. A tremendous job was done by the division of Parasitology, New England Biolabs, by mining the essential genes of *B. malayi*^23^. It provides a new gateway to develop and design new therapeutics for brugian filariasis. Unfortunately, *Wolbachia* (*Wol*) which is an excellent drug target for Lf was untouched. It is an obligate intracellular symbiotic bacterium of filarial nematodes. It plays very crucial role in the development, vitality and fertility of the filarial nematodes^24^,^25^. Targeting *Wol* would be very effective approach to monitor the human filariasis infections. In 2009, the first computational effort was made to identify the essential genes of the unculturable *Wolbachia* endosymbiotic bacterium^26^ in the lack of complete proteome of *Wol*. At present, we have complete genome of *Wolbachia* from *Brugia malayi* (*wBm*)^27^, which could be use to define the most suitable therapeutic targets in *wBm* through a hierarchical proteome subtractive approach. Several researchers have used this approach to identify the possible therapeutic targets^28^,^29^,^30^,^31^,^32^ in various pathogens. Therefore, we have adopted this approach with slight addition and modification to come up with possible therapeutic targets. The complete work flow of our approach has been demonstrated in Fig. 1. In this current approach, we have added estimation of the identified therapeutic targets through the druggability test and ensured that all identified drug targets are non-homologous to human proteins. Identified potential therapeutic targets were further modelled to get insight into their molecular organization and protein conformations.

All identified therapeutic targets in this study was modelled and stored in an open access database named FiloBase. In addition, we have modelled forty drug targets of *B. malayi* and incorporated in our database which was earlier identified by Kumar *et al.* from the England Biolabs^23^. In order to supplement and make it more informative, other significant filarial information such as ESTs sequences, potential epitopes, experimental drugs, experimental structures of nematode proteins was incorporated in FiloBase by extensive literature survey which was in demand to facilitate the drug discovery process of Lf^33^. At present, FiloBase contains 119 potential drug targets for Lf; we hope that FiloBase will be worthwhile to expedite the process of drug discovery for the better treatment of Lf.

## Materials and Methods

### Identification of pathogens metabolic pathways

Kyoto Encyclopedia of Genes and Genomes (KEGG) is a manually curated database which helps to understand the biological systems of the organisms. Metabolic pathway of *wBm* and human was retrieved from the KEGG database and manual comparison was performed to find out the unique and common pathways in pathogens. Metabolic pathways which were present in both (human and *wBm*) were considered as common pathways and those which were present only in *wBm* not in human host were identified as a unique pathway. Protein sequence of both the pathways were retrieved from the UniProt database and passed through the further proteome subtractive channel.

### Mining of non-homologous to human proteins of wBm

Retrieved protein sequences of *wBm* from both the pathways were subjected to BLASTp and sequence similarity search was performed against the human proteome database. The main objective of this step was to define the non-homologous to human proteins in *wBm*. It is likely to prevent the cross-reactivity of drug compounds with the human host proteins^34^. Here, we have used ‘Expect’ value (e-value) <0.005 and a minimum bit score of >100 to exclude the homologous sequences. Proteins which showed “HITS” with the above mentioned cut-off values were considered as non-homologous proteins^35^,^36^,^37^ and carried for further screening process, while remaining sequences were excluded from the list.

### Essentiality assessment of *wBm* proteins

In order to identify the essential proteins of *wBm*, resultant non-homologous to human protein sequences of *wBm* were subjected to the protein BLAST tool and similarity search was performed against the essential protein sequence of bacteria from Database of Essential Gene (DEG)^38^ with an e-value <0.0001 and bit score >100^39^. DEG is a database which contains indispensable genes from bacteria, archaea, and eukaryote organisms which support their cellular life. Currently, it holds 12,926 essential genes of bacteria. Based on the assumptions that similar proteins which are essential in one bacteria may be essential for another bacteria, hits found with DEG database with the above mentioned cut-off values were expected to represent the crucial proteins of *wBm* while remaining proteins were not, therefore excluded from the list of probable drug target.

### Drug prioritization

All these non-homologous to human and essential proteins of *wBm* can be treated as potential therapeutic targets. However, being non-homologous to human and indispensable for the pathogen survival is not only the criteria to identify the most suitable drug targets for any pathogens, other vital parameters such as low molecular weight, sub-cellular localizations and their ability to interact with potential drugs (druggability) are also playing very significant role to identify the potential drug targets. Therefore, screening of potential therapeutic targets of *wBm* were carried out by identifying their biological significance and sub-cellular localization using Gneg-mPLoc^40^. Gneg-mPLoc is a powerful tool to identify the sub-cellular location of gram negative bacteria proteins. It identifies the gram negative protein localization in the following eight locations (1) cytoplasm, (2) extracellular, (3) fimbrium, (4) flagellum, (5) inner membrane, (6) nucleoid, (7) outer membrane and (8) periplasm^40^. Resultant data sets of Gneg-mPLoc were further cross-checked with CELLO v.2.5^41^ and PSORTb II^42^ which are another web based tool for the prediction of sub-cellular localization of protein. The main objective of this study was to classify the proteins as potential drugs and vaccine targets. The cytoplasmic and inner membrane proteins were considered as potential drug targets, whereas the surface membrane, peri-plasmic and extracellular proteins were considered as potential vaccine targets. Prevention from the Lf can be accomplished through the vaccine development which involves antigenic surfaces to trigger the humoral immune responses. Therefore, membrane associated protein candidates are likely to represents as potential therapeutic targets to develop new vaccine, epitopes and as well as powerful drug candidates.

### Druggability analysis

The most reliable way to identify the druggability of a protein is to identify the similar protein which binds to the drug-like compound^43^,^44^. Therefore, all identified potential drug targets were further evaluated based on the druggability test. In this step, all identified potential drug targets were searched against the DrugBank database^45^ and TTD database^46^. For this study, we have downloaded all FDA approved drugs (1,478), FDA approved Biotech drugs (183), experimental drugs (2,718), approved enzymes (174) and approved transporters (79) from DrugBank and TTD database (2134). Hits found with DrugBank and TTD database was considered as druggable targets while remaining were considered as novel drug targets which further need to be validated experimentally.

### Tertiary structure identification

The tertiary structures of identified possible therapeutic and vaccine targets were searched in the PDB^47^ database for experimental structure. Available structures were incorporated in our database, while, un-available structures were searched for suitable templates for building their tertiary structure using NCBI protein BLAST tool. Template structures which have shown sequence identity more than 40% and query coverage more than 90% were modelled using Modeller9v10^48^ and sequence which exhibits lower sequence identities with template proteins were modelled using I-TASSER (Iterative Threading ASSEmbly Refinement) server^49^ or Phyre2 (Protein Homology/analogY Recognition Engine) server^50^. I-TASSER builds a 3D model of the user given protein sequences based on the multiple threading approach through LOMETS (Local Meta-Threading Server) and iterative template fragment assembly^51^ while Phyre2 predicts protein structure based on the remote homology recognition techniques. All the modelled structures were verified using Structural Analysis and Verification Server (SAVeS) for PROCHECK^52^ to evaluate their stereo-chemical quality by analyzing residue-by-residue geometry and ERRAT^53^ was used to evaluate their overall structural quality. PROVE program was also used to verify our modelled structure. It verifies the structure based on the Z-score deviation.

### Database development and organization

**Figure 2:**
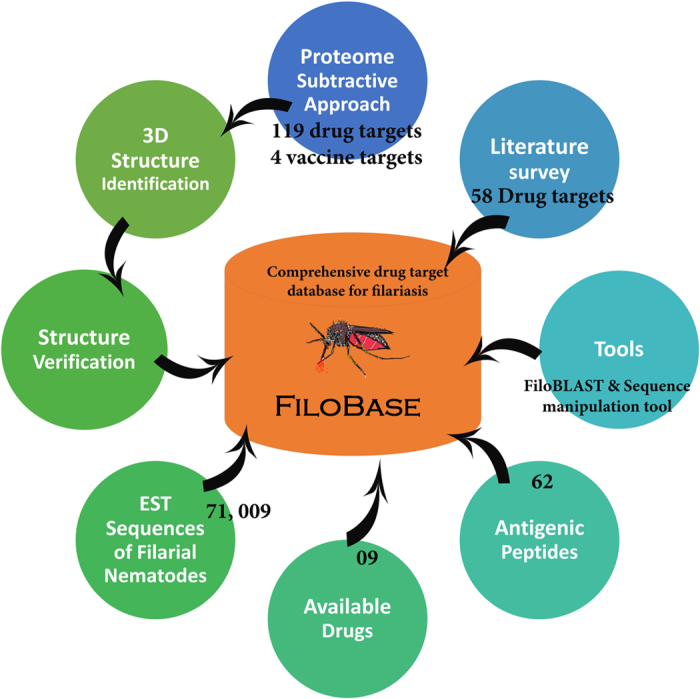
Architecture of the FiloBase database.

All identified potential drug targets were stored in a user friendly open access database named FiloBase. All data were stored in a My-SQL and hosted using an Apache server. The complete flow-chart of the database development was illustrated in Fig. 2. The user interface of the database was prepared in PHP and JavaScript whereas; back-end was supported by PHP and Bio-Perl. The entire database contents was classified in five different search categories, (1) Drug targets of *B. malayi* and *W. bancrofti*, (2) EST sequences of *B. Malayi, B. timori, B. Pahangi* and *Wol*, (3) Epitope sequences from extensive literature survey, (4) Available and potential drug compounds with literature support and (5) Potential vaccine targets for *wBm*.

**Figure 3:**
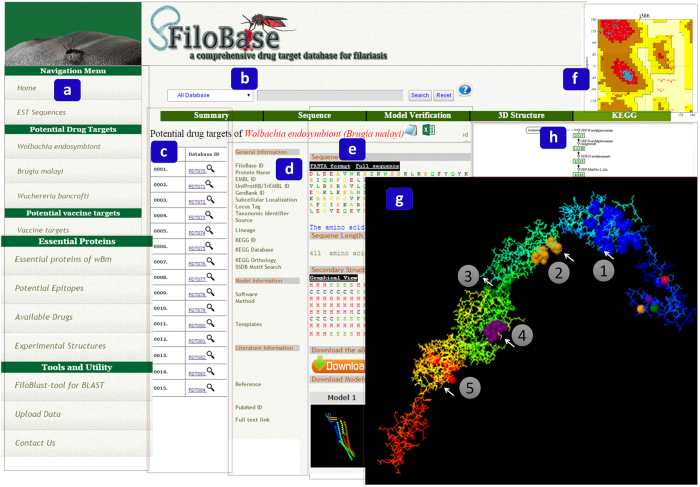
The snapshot of the “Home Page” and “Result Page” of the FiloBase. (**a**) Navigation window: It includes direct link to the various classified data in a single click, (**b**) Query search through text query, (**c**) List of potential drugs for *Wolbachia* endosymbiont, (**d**) *Summary tab* of the potential drug targets: It consists brief information about the therapeutic target, (**e**) *Sequence tab*: it contains the sequence and chain information of target protein, (**f**) *Model verification tab*: It contains model verification results obtained from PROCHECK, ERRAT and PROVE, (**g**) *3D structure tab*: It contains the 3D modelled structure of the target protein with their active site pockets and (**h**) *KEGG tab*: It contains the graphical output of KEGG pathways if available.

User can easily fetch desired information by selecting the query field and enter their query keywords in the given text box from the home page of the database. To make our retrieval system user friendly and more convenient, we have classified all the information and made available by a click on the table menu on the home page of the database (Fig. 3a).

## Results and Discussion

### Identification of metabolic pathways for *wBm*

Here, we report first computational approach to identify potential therapeutic targets of *wBm* by the protein subtractive approach. It was proved as a successful approach to identify the potential drug target proteins which are involved in various metabolic pathways of pathogen, but absent in the host organism and essential for their survival^54^. Overall summary of the project is depicted in the Table 1. In this current study, we have considered several vital parameters and systematic approach with a drug prioritization method to come with superior drug and vaccine targets. Thus, identified drug targets supposed to exhibits less side-effect and may signify as a supreme drug or a vaccine targets for the treatment of Lf.

Initially, metabolic pathway information of *wBm* and human host was collected from the KEGG database. At present, KEGG contains 281 metabolic pathways of human and 65 metabolic pathways of *wBm*. Following our protocol, we identified 5 unique metabolic pathways and 44 common metabolic pathways of *wBm* (Supplementary Table 1). Protein sequences from unique (50) and common metabolic pathways (460) of *wBm* were retrieved from the UniProt database and submitted to NCBI Protein BLAST, against the human. It results 156 proteins that showed “NO HITS” against the human proteome, remaining 311 proteins were excluded from the lists.

### Essential proteins of *wBm*

In order to identify the essentiality of 156 non-homologous protein sequence of *wBm,* a sequence similarity searched was performed against the DEG database using protein BLAST tool. It resulted, 115 proteins, among these, 24 *wBm* proteins were belonging to the unique metabolic pathways and 91 proteins were from common metabolic pathways (Supplementary Table 2). These 115 proteins were likely to represents the significant role in metabolic pathways of *wBm*, non-homologous to human and indispensable for their survival. Further we identified the length of the essential proteins. These initial target protein lists can be used as a potential drug target; however, being non-homologous to human, essential and their involvement in various metabolic pathways is not an only criterion for the selection of potential drug targets. Therefore, to minimize the drug side-effects and for the identification of most potential drug targets of *wBm*, we further investigated these drug targets for low molecular weight, sub-cellular localization, experimental structure and druggability parameters. Through low molecular weight analysis we identified 10 protein sequences which have sequence length less than 100 kD. As it has been reported earlier by Dutta *et. al*. that short length sequences have less chance to represent an attractive drug target^55^, we screened this short length protein sequences from the potential drug target list. It results 105 protein sequences.

### Prioritization of essential non-homologous proteins of *wBm*

Since, sub-cellular localization of protein plays a major role to understand the protein functions which could be essential for drug discovery and development process^56^. To investigate the sub-cellular localization of these 105 protein sequences; Gneg-mPLoc was used and resultant data was crosschecked with CELLO server. The main objective of this step is to identify the locations of the proteins in the cell which helps to differentiate between the drug and vaccine targets^32^. Protein founds in the cytoplasmic, periplasmic or inner-membrane region was considered as potential drug targets while extracellular proteins were recognized as potential vaccine targets. We identified 101 potential drug targets (Supplementary Table 3) and 4 potential vaccine targets (Table 2).

### Druggability of essential non-homologous proteins of wBm

The druggability of essential *wBm* proteins were evaluated based on the assumptions that a druggable protein targets should interact with drug-like compound^57^,^58^. For this reason, each essential, non-homologous protein of *wBm* which plays a key role in metabolic pathway were accessed in the standalone BLASTp tool to find the similar drug target homology against the DrugBank and TTD database with an e-value less than 0.005. Proteins which showed hits with the defined cut-off values were recognized as significant homologs^59^ whereas, remaining proteins were not therefore excluded from the future study (Supplementary Table 4). Out of 101, we identified 61 drug targets which have shown similarity with the drug targets available in DrugBank and TTD database. Interestingly, 56 drug targets from DrugBank and 25 drug targets from TTD database have shown similarity, among these 20 drug targets were common in both the database. Ultimately, we identified 61 highly potential druggable targets (Table 3) and 4 potential vaccine targets (Table 2). These proposed therapeutic targets can be utilized for the further experimental studies to develop the most anti *wBm* therapeutics to treat the Lf.

### Data compilation and database development

Identified drug targets and vaccine targets were stored in a user friendly database and we named it as FiloBase-a comprehensive drug target database for filariasis. The snapshot of the home page and the result page of FiloBase have been shown in Fig. 3.

The inaugural release of FiloBase contains a total no. of 119 potential drug targets of filarial nematodes. We have collected 58 drug targets of *B. malayi* from the literature survey^23^,^33^ and 61 drug targets from our current study through subtractive proteome approach for *wBm*. Since, structural information plays an important role for the drug and vaccine development^60^. These drug targets were stored in our database which can be fetched through the classified tab options present in the left-side of the database page (Fig. 3a). User can also enter their keywords to the query box available at the home page of the database and retrieve desired information. It will fetch the drug target information, related to the user given query (Fig. 3b).

All identified drug targets were modelled for their structural information using Modeller9v11, Phyre2.0 and I-TASSER server. Reliability of modelled drug targets was further evaluated using PROCHECK, ERRAT and PROVE server. The verification data of the modelled structure are stored in the model verification tab of the result page of the potential drug targets. User can fetch the desire information from the given link and can further improve the quality of the modelled structure if needed. All verified structures were incorporated in our database with additional information about potential drug targets to render it more enlightening and useful. Possible binding site residues were identified based on the template proteins and structure was shown three dimensionally using JSmol visualize.

In sequence tab, solvent accessibility and secondary structure were shown for each potential drug targets. User can get the graphical view of the secondary structure through the link given at the result page of the database. Alignment file of target and template proteins is also given in the result page to database to crosscheck the quality of the modelled structure (Fig. 3). This information may be useful for further development and to get insight into the structural level of the potential therapeutic targets.

In 3D Structure tab, a user friendly JSmol visualizer tool was used to visualize the modelled structure of therapeutics targets. In addition, based on the template, we have identified possible binding sites and listed in the result page. User can click on the active site pockets to explore the binding pockets and get insight into their positions and contributing residues using JSmol button (Fig. 3f). In KEGG tab, we have shown the role of the corresponding drug target in the metabolic pathway (Fig. 3g).

To make our database more informative, we have cross-linked our database with various related databases such as KEGG database for Pathways analysis, sequence similarity database in KEGG (SSDB) for motif search, UniProt and EMBL database for sequence information.

Various literatures^33^,^61^,^62^,^63^, databases and various websites [World Health Organization (WHO), Centre for Disease Control and Preventions (CDC), National Vector Borne Disease Control Programme-India (NVBDCP), DrugBank and Drugs.com (http://www.drugs.com/), Medscape (http://emedicine.medscape.com/), GAELF (http://www.filariasis.org/), The carter center (http://www.cartercenter.org/health/lf/)] were mined in the hunt of available drugs for the treatment of Lf and incorporated in our database with their 3D structure information. Drugs available in various brand names have been also collected with their general descriptions, clinical pharmacology, pharmacokinetics, contraindications, warnings, precautions and dosage and administration information data. Entire drug candidates are linked to DrugBank, KEGG and PubChem database for further information.

In addition, we have collected 71,009 EST sequences from various filariasis causing nematodes [*B. malayi* (65,536)*, B. pahangi* (318)*, B. timori* (26)*, W. bancrofti* (5103) and *wBm* (26)] and stored in our database. These EST sequences of filariasis causing nematodes and *wBm* bacteria with add-on information will be useful for various groups of researchers.

Due to the advancements in biological sciences, an enormous number of sequence data were generated. Therefore, BLAST has become essential tool for biologists to compare these proteins and nucleotide sequences with largest set of datasets to investigate the similarities between them. For that reason, we have developed a standalone BLAST tool for filariasis and named it FiloBLAST. Query sequences can be entered in the single letter amino acid or in nucleotide code and based on the input sequence type algorithm can be selected. At present, user can BLAST their query sequences against the local copy of EST databases, Protein databases and drug targets from DrugBank databases. For EST databases, we have collected EST sequences from all the filariasis causing nematodes and made a local copy for BLAST search. We have also downloaded EST sequences from NEMBASE4^64^ and given a link to the NEMBASE4 database for further information. Likewise, we have also collected data from Protein Data Bank^65^, Swiss-Prot^66^, and DrugBank^67^ database and made a local copy so that user can query their nucleotide and protein sequences against these databases to fetch the desired information in a single platform.

For potential and experimentally proved epitope sequences of filariasis causing nematodes were identified through the enormous literature survey and databases such as PubMed and Google Scholar. Various epitope databases were queried (IEDB^68^, BCIpep^69^ and SEDB^58^) to recognize the experimental and potential epitopes. At present, FiloBase contains 62 epitopes; 27 from *B. malayi* and 35 from *W. bancrofti*. A quick link has been provided in the home page of the database to fetch this information in a single click. We will timely update our database as earliest filarial data will be reported in the literature or authentic web resources. We have further provided an online data submission tool for users to upload their data in our database. Submitted data will be verified and may incorporate into our database. User can send their suggestions for further improvement of FiloBase.

## Conclusions

In summary, owing to the emergence of resistant and limitations of access drugs for Lf, current research interest is focused on the identification of novel drug and vaccine targets to enhance the treatment of Lf. Using comparative proteome subtractive approach, we have pruned those drug targets which may be less effective or may cause severe side-effects. Therefore, proteins which are indispensable to the survival of pathogens and non-homologous to host proteins were considered as potential therapeutic targets. Druggability approach was further applied to the potential drug target data sets to expose the more probable drug targets. Our investigation reveals 61 potential drug targets and 4 potential vaccine target for *wBm* which could be further validated experimentally through the drug and vaccine design pipelines. Identified drug and vaccine targets were further modelled and then verified for their structural quality. Sequentially, we have enriched these data with essential information which may enlighten and support further research on *wBm*. All information was stored in a single stop web based platform. We named it FiloBase. We have also collected filariasis related information and records from various literatures and authenticated website to make accessible for the filarial researcher community through a user friendly database. In future, our database can be hereby extended by identifying potential drug targets from other filarial causing nematodes.

## Additional Information

**How to cite this article**: Sharma, O. P. and Kumar, M. S. Essential proteins and possible therapeutic targets of *Wolbachia* endosymbiont and development of FiloBase-a comprehensive drug target database for Lymphatic filariasis. *Sci. Rep.* **6**, 19842; doi: 10.1038/srep19842 (2016).

## Supplementary Material

Supplementary Information

## Figures and Tables

**Figure 1:**
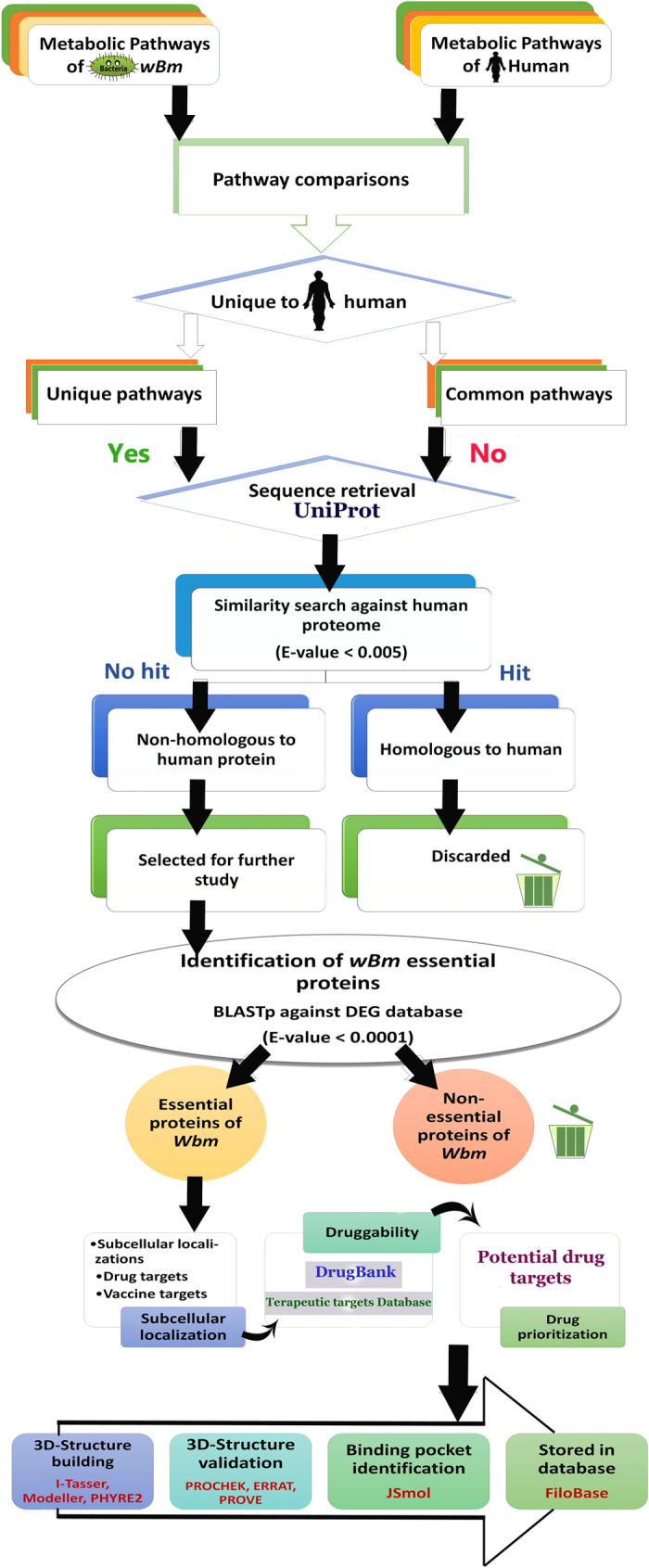
Schematic representation of the various steps involved to identify the potential therapeutic targets and vaccine targets of *wBm* through extensive proteome subtractive approached.

**Table 1:** Overall statistics of the subtractive proteome approach for drug target identification of the *wBm*.

Unique Pathway Proteins (UPP) Analysis	Proteins In Numbers	Common Pathway Protein (CPP) Analysis	Proteins In Numbers
Unique pathway proteins (UPP)	50	Common pathway proteins (CPP)	460
Excluded repeated proteins from CPP	01	Excluded repeated proteins from CPP	49
		Excluded common proteins between CPP and UPP	14
		Total proteins	397
Homologous to human	13	Homologous to human	298
Non-homologous to human	37	Non-homologous to human	119
Non-Homologous To Human Proteins From UPP And CPP			
Total Non-homologous protein sequences		156	
Essential proteins (Homologous to DEG)		115	
Protein sequence less than 100 amino acid		10 [115−10 = 105]	
Subcellular localization [Gneg-mPLoc]			
Drug targets [Cytoplasm, Inner membrane, Periplasm]		101 [75 + 22 + 4 = 101 ]	
Vaccine targets [Extracellular]		4	
Druggability			
DrugBank		56	
TTD		25	
Common between DrugBank and TTD		20	
Total druggable targets		61	
Total potential druggable drug targets of *wBm*		61	

**Table 2:** Identified potential vaccine targets of *wBm*.

Sl.No	FiloBase ID	UniProt ID	Gene Name	Subcellular Localization	KEGG Pathways	Pathways Name
1	FVT001	Q5GTG1	Putative peptidoglycan lipid II flippase	Extra cellular	*Wbm*0122	*Wbm00550* Peptidoglycan biosynthesis
2	FVT002	Q5GSZ3	Deoxycytidine triphosphate deaminase	Extra cellular	*Wbm*0292	*Wbm*00240 Pyrimidine metabolism *Wbm*01100 Metabolic pathways
3	FVT003	Q5GT07	GTP cyclohydrolase II	Extra cellular	*Wbm*0278	*Wbm*00740 Riboflavin metabolism *Wbm*01100 Metabolic pathways
4	FVT004	Q5GT39	RNA pyrophosphohydrolase	Extra cellular	*Wbm*0246	*Wbm*03018 RNA degradation

**Table 3:** Identified potential therapeutic targets of *wBm.*

Sl. No.	FiloBase ID	UniProt	Protein Name	Sl. No.	FiloBase ID	UniProt	Protein Name
1.	FDT070	Q5GS61	Outer membrane protein	32.	FDT102	Q5GTJ9	Riboflavin synthase alpha chain
2.	FDT071	Q5GTP1	N5-carboxyaminoimidazole ribonucleotide synthase	33.	FDT103	Q5GTK4	Holo-[acyl-carrier-protein] synthase
3.	FDT072	Q5GRP6	UDP-N-acetylglucosamine 1-carboxyvinyltransferase	34.	FDT104	Q5GRR7	Ferrochelatase
4.	FDT073	Q5GRK8	UDP-N-acetylenolpyruvoylglucosamine reductase	35.	FDT105	Q5GTL0	3-polyprenyl-4-hydroxybenzoate decarboxylase
5.	FDT074	Q5GSC8	UDP-N-acetylmuramoylalanine—D-glutamate ligase	36.	FDT106	Q5GTN6	4-hydroxy-3-methylbut-2-enyl diphosphate reductase
6.	FDT075	Q5GS66	D-alanine—D-alanine ligase	37.	FDT107	Q5GSU3	30S ribosomal protein S10
7.	FDT076	Q5GT47	UDP-N-acetylmuramoyl-tripeptide—D-alanyl-D-alanine ligase	38.	FDT108	Q5GSV0	30S ribosomal protein S3
8.	FDT077	Q5GTK7	Cell division protein FtsI	39.	FDT109	Q5GSV8	30S ribosomal protein S8
9.	FDT078	Q5GSZ5	D-alanyl-D-alanine carboxypeptidase	40.	FDT111	Q5GS93	DNA polymerase III epsilon subunit
10.	FDT079	Q5GT03	Type IV secretory pathway;VirB11 component	41.	FDT112	Q5GSK2	DNA polymerase III;gamma/tau subunit
11.	FDT080	Q5GT10	Glutamine synthetase	42.	FDT113	Q5GSK7	DNA polymerase III subunit beta
12.	FDT081	Q5GTF5	Membrane associated signal transduction histidine kinase	43.	FDT114	Q5GSY6	Replicative DNA helicase;DnaB
13.	FDT082	Q5GT99	PleD-like response regulator containing 2 CheY-like receiver domains and a GGDEF domain	44.	FDT115	Q5GSN3	independent phosphoglycerate mutase
14.	FDT083	Q5GRI5	Phosphopantetheine adenylyltransferase	45.	FDT116	Q5GTG5	UDP-N-acetylmuramate—L-alanine ligase
15.	FDT084	Q5GTH4	Dihydrolipoyllysine-residue succinyltransferase component of 2-oxoglutarate dehydrogenase complex	46.	FDT117	Q5GSE4	UDP-N-acetylmuramoyl-L-alanyl-D-glutamate—2
16.	FDT085	Q5GTJ4	Bifunctional protein GlmU	47.	FDT118	Q5GS79	UDP-N-acetylglucosamine—N-acetylmuramyl-(pentapeptide) pyrophosphoryl-undecaprenol N-acetylglucosamine transferase
17.	FDT086	Q5GSI1	Acetyl/propionyl-CoA carboxylase;alpha subunit	48.	FDT119	Q5GS12	NADH-quinone oxidoreductase subunit K
18.	FDT087	Q5GSM8	Triosephosphate isomerase	49.	FDT120	Q5GS13	NADH:ubiquinone oxidoreductase chain L
19.	FDT088	Q5GSF0	Succinate dehydrogenase subunit C sdhC	50.	FDT121	Q5GTP0	Aspartate-semialdehyde dehydrogenase
20.	FDT089	Q5GSX1	ATP synthase subunit alpha	51.	FDT122	Q5GTQ6	Riboflavin biosynthesis protein RibD
21.	FDT090	Q5GS22	3-oxoacyl-[acyl-carrier-protein] synthase 3	52.	FDT123	Q5GTA4	1-deoxy-D-xylulose 5-phosphate reductoisomerase
22.	FDT091	Q5GRP7	3-oxoacyl-[acyl-carrier-protein] synthase 2	53.	FDT124	Q5GTB0	4-diphosphocytidyl-2-C-methyl-D-erythritol kinase
23.	FDT092	Q5GTN0	3-hydroxyacyl-[acyl-carrier-protein] dehydratase FabZ	54.	FDT125	Q5GRY7	50S ribosomal protein L10
24.	FDT093	Q5GRN3	Probable trans-2-enoyl-ACP reductase;FabK	55.	FDT126	Q5GRM6	Signal peptidase I
25.	FDT094	Q5GSN9	N5-carboxyaminoimidazole ribonucleotide mutase	56.	FDT127	Q5GTF9	Single-stranded DNA-specific exonuclease RecJ
26.	FDT095	Q5GSW7	DNA-directed RNA polymerase subunit alpha	57.	FDT128	Q5GTA1	NAD-specific glutamate dehydrogenase
27.	FDT096	Q5GRJ9	Orotidine 5′-phosphate decarboxylase	58.	FDT129	Q5GSL1	Phosphatidylserine synthase
28.	FDT098	Q5GTA6	4-hydroxy-tetrahydrodipicolinate reductase	59.	FDT130	Q5GS87	5′-nucleotidase SurE
29.	FDT099	Q5GSI7	2,3,4,5-tetrahydropyridine-2,6-dicarboxylate N-succinyltransferase	60.	FDT132	Q5GSX3	3;4-dihydroxy-2-butanone 4-phosphate synthase
30.	FDT100	Q5GS70	Thioredoxin reductase	61.	FDT133	Q5GRI7	Lysine—tRNA ligase
31.	FDT101	Q5GT94	6;7-dimethyl-8-ribityllumazine synthase				
