# Supplementary material for: Essential proteins and possible therapeutic targets of Wolbachia endosymbiont and development of FiloBase-a comprehensive drug target database for Lymphatic filariasis
Source: Sci Rep. 2016 Jan 25;6:19842. doi: 10.1038/srep19842 (PMC4726333; doi:10.1038/srep19842)
Supplement: Supplementary Information [file srep19842-s1.doc]

**Essential proteins and possible therapeutic targets** **of *Wolbachia* endosymbiont and development of FiloBase-a comprehensive drug target database for Lymphatic filariasis**

Om Prakash Sharma^1^ and Muthuvel Suresh Kumar^1^*****

^1^Centre for Bioinformatics, School of Life Science, Pondicherry University, Pondicherry-605014, India

**SUPPLEMENTARY TABLE LEGENDS**

**Supplementary Table 1**

Unique and common metabolic pathways of *wBm* with reference to the human metabolic pathways.

**Supplementary Table 2**

Essential and non-homologous to human proteins of *wBm* obtained from KEGG pathways.

**Supplementary Table 3**

Potential drug targets of *wBm.*

**Supplementary Table 4:** Druggable therapeutic targets of *wBm.* Druggability was analyzes against the DrugBank database and TTD database result.

**Supplementary Table 1:** Unique and common metabolic pathways of *wBm* with reference to the human metabolic pathways.

| **UNIQUE PATHWAY PROTEINS** | | | |
| --- | --- | --- | --- |
| **Sl. No.** | **Pathway ID** | **Pathway name** | **Number of proteins** |
| **1** | 00680 | Methane metabolism | 06 |
| **2** | 00550 | Peptidoglycan biosynthesis | 12 |
| **3** | 03070 | Bacterial secretion system | 20 |
| **4** | 02020 | Two-component system | 10 |
| **5** | 00312 | Beta-Lactam resistance | 2 |
|  | | | **Total: 50** |
| **COMMON PATHWAY PROTEINS** | | | |
| **1** | 00010 | Glycolysis / Gluconeogenesis | 10 |
| **2** | 00020 | Citrate cycle (TCA cycle) | 13 |
| **3** | 00030 | Pentose phosphate pathway | 06 |
| **4** | 00051 | Fructose and mannose metabolism | 4 |
| **5** | 00520 | Amino sugar and nucleotide sugar metabolism | 06 |
| **6** | 00620 | Pyruvate metabolism | 7 |
| **7** | 00630 | Glyoxylate and dicarboxylate metabolism | 7 |
| **8** | 00640 | Propanoate metabolism | 3 |
| **9** | 00562 | Inositol phosphate metabolism | 1 |
| **10** | 00190 | Oxidative phosphorylation | 38 |
| **11** | 00910 | Nitrogen metabolism | 2 |
| **12** | 00920 | Sulfur metabolism | 1 |
| **13** | 00061 | Fatty acid biosynthesis | 8 |
| **14** | 00561 | Glycerolipid metabolism | 7 |
| **15** | 00564 | Glycerophospholipid metabolism | 7 |
| **16** | 00230 | Purine metabolism | 30 |
| **17** | 00240 | Pyrimidine metabolism | 23 |
| **18** | 00250 | Alanine, aspartate and glutamate metabolism | 10 |
| **19** | 00260 | Glycine, serine and threonine metabolism | 7 |
| **20** | 00270 | Cysteine and methionine metabolism | 6 |
| **21** | 00280 | Valine, leucine and isoleucine degradation | 2 |
| **22** | 00300 | Lysine biosynthesis | 9 |
| **23** | 00310 | Lysine degradation | 5 |
| **24** | 00330 | Arginine and proline metabolism | 5 |
| **25** | 00350 | Tyrosine metabolism | 2 |
| **26** | 00400 | Phenylalanine, tyrosine and tryptophan biosynthesis | 1 |
| **27** | 00450 | Selenocompound metabolism | 3 |
| **28** | 00471 | D-Glutamine and D-glutamate metabolism | 2 |
| **29** | 00480 | Glutathione metabolism | 5 |
| **30** | 00740 | Riboflavin metabolism | 8 |
| **31** | 00760 | Nicotinate and nicotinamide metabolism | 2 |
| **32** | 00770 | Pantothenate and CoA biosynthesis | 3 |
| **33** | 00780 | Biotin metabolism | 5 |
| **34** | 00785 | Lipoic acid metabolism | 2 |
| **35** | 00670 | One carbon pool by folate | 8 |
| **36** | 00860 | Porphyrin and chlorophyll metabolism | 10 |
| **37** | 00130 | Ubiquinone and other terpenoid-quinone biosynthesis | 09 |
| **38** | 00900 | Terpenoid backbone biosynthesis | 10 |
| **39** | 03020 | RNA polymerase | 3 |
| **40** | 03010 | Ribosome | 53 |
| **41** | 00970 | Aminoacyl-tRNA biosynthesis | 20 |
| **42** | 03060 | Protein export | 14 |
| **43** | 04122 | Sulfur relay system | 4 |
| **44** | 03018 | RNA degradation | 9 |
| **45** | 03030 | DNA replication | 13 |
| **46** | 03410 | Base excision repair | 10 |
| **47** | 03420 | Nucleotide excision repair | 7 |
| **48** | 03430 | Mismatch repair | 7 |
| **49** | 03444 | Homologous recombination | 13 |
| **50** | 02010 | ABC transporters | 20 |
|  | | | **Total:**460 |

**Supplementary Table 2:** Essential and non-homologous to human proteins of *wBm* obtained from KEGG pathways.

| **Sl. No.** | **UniProt ID** | **KEGG entry** | **Sub-cellular Localization** | **Seq Length** | **TMHMM** | **Protein name** | **Pathways name and ID** |
| --- | --- | --- | --- | --- | --- | --- | --- |
| 1. **1** | Q5GTC5 | Wbm0158 | Cytoplasm |  | No | Fructose 1,6-bisphosphatase II (EC:3.1.3.11) | wbm00010 Glycolysis / Gluconeogenesis  wbm00030 Pentose phosphate pathway  wbm00051 Fructose and mannose metabolism  wbm00680 Methane metabolism  wbm01100 Metabolic pathways  wbm01110 Biosynthesis of secondary metabolites  wbm01120 Microbial metabolism in diverse environments  wbm01200 Carbon metabolism |
|  | Q5GSN3 | Wbm0403 | Cytoplasm | 501 | No | 2,3-bisphosphoglycerate-independent phosphoglycerate mutase [EC:[5.4.2.12](http://www.genome.jp/dbget-bin/www_bget?ec:5.4.2.12)] | wbm00010 Glycolysis / Gluconeogenesis  wbm00260 Glycine, serine and threonine metabolism  wbm00680 Methane metabolism  wbm01100 Metabolic pathways  wbm01110 Biosynthesis of secondary metabolites  wbm01120 Microbial metabolism in diverse environments  wbm01200 Carbon metabolism  wbm01230 Biosynthesis of amino acids |
|  | Q5GRP6 | Wbm0740 | Inner-Membrane | 425 |  | UDP-N-acetylglucosamine 1-carboxyvinyltransferase [EC:[2.5.1.7](http://www.genome.jp/dbget-bin/www_bget?ec:2.5.1.7)] | wbm00520 Amino sugar and nucleotide sugar metabolism  wbm00550 Peptidoglycan biosynthesis  wbm01100 Metabolic pathways |
|  | Q5GRK8 | Wbm0778 | Inner-Membrane | 295 |  | UDP-N-acetylmuramate dehydrogenase [EC:[1.3.1.98](http://www.genome.jp/dbget-bin/www_bget?ec:1.3.1.98)] | wbm00520 Amino sugar and nucleotide sugar metabolism  wbm00550 Peptidoglycan biosynthesis  wbm01100 Metabolic pathways |
|  | Q5GTG5 | Wbm0118 | Cytoplasm | 536 |  | UDP-N-acetylmuramate--alanine ligase [EC:[6.3.2.8](http://www.genome.jp/dbget-bin/www_bget?ec:6.3.2.8)] | wbm00471 D-Glutamine and D-glutamate metabolism  wbm00550 Peptidoglycan biosynthesis  wbm01100 Metabolic pathways |
|  | Q5GSC8 | Wbm0508 | Cytoplasm | 498 |  | UDP-N-acetylmuramoylalanine--D-glutamate ligase [EC:[6.3.2.9](http://www.genome.jp/dbget-bin/www_bget?ec:6.3.2.9)] | wbm00471 D-Glutamine and D-glutamate metabolism  wbm00550 Peptidoglycan biosynthesis  wbm01100 Metabolic pathways |
|  | Q5GS66 | Wbm0570 | Cytoplasm | 339 | Yes | D-alanine-D-alanine ligase [EC:[6.3.2.4](http://www.genome.jp/dbget-bin/www_bget?ec:6.3.2.4)] | wbm00550 Peptidoglycan biosynthesis  wbm01100 Metabolic pathways |
|  | Q5GSE4 | Wbm0492 | Cytoplasm | 496 |  | UDP-N-acetylmuramoyl-L-alanyl-D-glutamate--2,6-diaminopimelate ligase [EC:[6.3.2.13](http://www.genome.jp/dbget-bin/www_bget?ec:6.3.2.13)] | wbm00300 Lysine biosynthesis  wbm00550 Peptidoglycan biosynthesis |
|  | Q5GT47 | Wbm0238 | Cytoplasm | 455 |  | UDP-N-acetylmuramoyl-tripeptide--D-alanyl-D-alanine ligase [EC:[6.3.2.10](http://www.genome.jp/dbget-bin/www_bget?ec:6.3.2.10)] | wbm00300 Lysine biosynthesis  wbm00550 Peptidoglycan biosynthesis  wbm01100 Metabolic pathways |
|  | Q5GS79 | Wbm0557 | Cytoplasm | 343 | Yes | UDP-N-acetylglucosamine--N-acetylmuramyl-(pentapeptide) pyrophosphoryl-undecaprenol N-acetylglucosamine transferase [EC:[2.4.1.227](http://www.genome.jp/dbget-bin/www_bget?ec:2.4.1.227)] | wbm00550 Peptidoglycan biosynthesis  wbm01100 Metabolic pathways |
|  | Q5GTG1 | Wbm0122 | Extracellular | 497 | Yes | Putative peptidoglycan lipid II flippase | wbm00550 Peptidoglycan biosynthesis |
|  | Q5GTK7 | Wbm0075 | Inner-membrane | 521 |  | Penicillin-binding protein 2 | wbm00312 Beta-Lactam resistance  wbm00550 Peptidoglycan biosynthesis |
|  | Q5GSZ5 | Wbm0290 | Cytoplasm | 370 |  | D-alanyl-D-alanine carboxypeptidase (penicillin-binding protein 5/6) [EC:[3.4.16.4](http://www.genome.jp/dbget-bin/www_bget?ec:3.4.16.4)] | wbm00550 Peptidoglycan biosynthesis  wbm01100 Metabolic pathways |
|  | Q5GT97 | Wbm0186 | Inner-membrane | 293 | Yes | Preprotein translocase subunit SecF | wbm03060 Protein export  wbm03070 Bacterial secretion system |
|  | Q5GSW3 | Wbm0322 | Inner-membrane | 442 | Yes | Preprotein translocase subunit SecY | wbm03060 Protein export  wbm03070 Bacterial secretion system |
|  | Q5GT19 | Wbm0266 | Inner-membrane | 867 |  | Preprotein translocase subunit SecA | wbm03060 Protein export  wbm03070 Bacterial secretion system |
|  | Q5GS95 | Wbm0541 | Cytoplasm | 167 |  | Preprotein translocase subunit SecB | wbm03060 Protein export  wbm03070 Bacterial secretion system |
|  | Q5GT61 | Wbm0222 | Cytoplasm | 144 | Yes | Preprotein translocase subunit YajC | wbm03060 Protein export  wbm03070 Bacterial secretion system |
|  | Q5GSY8 | Wbm0297 | Inner-membrane | 255 | Yes | Sec-independent protein translocase protein TatC | wbm03060 Protein export  wbm03070 Bacterial secretion system |
|  | Q5GT03 | Wbm0282 | Inner-membrane | 335 |  | Type IV secretion system protein VirB11 | wbm03070 Bacterial secretion system |
|  | Q5GT09 | Wbm0276 | Cytoplasm | 460 |  | Chromosomal replication initiator protein | wbm02020 Two-component system |
|  | Q5GT10 | Wbm0275 | Cytoplasm | 242 |  | Glutamine synthetase [EC:[6.3.1.2](http://www.genome.jp/dbget-bin/www_bget?ec:6.3.1.2)] | wbm00250 Alanine, aspartate and glutamate metabolism  wbm00330 Arginine and proline metabolism  wbm00630 Glyoxylate and dicarboxylate metabolism  wbm00910 Nitrogen metabolism  wbm01100 Metabolic pathways  wbm01120 Microbial metabolism in diverse environments  wbm01230 Biosynthesis of amino acids  wbm02020 Two-component system |
|  | Q5GTF5 | Wbm0128 | Inner-membrane | 475 | Yes | Two-component system, cell cycle sensor histidine kinase PleC [EC:[2.7.13.3](http://www.genome.jp/dbget-bin/www_bget?ec:2.7.13.3)] | wbm02020 Two-component system |
|  | Q5GT99 | Wbm0184 | Cytoplasm | 448 |  | Two-component system, cell cycle response regulator | wbm02020 Two-component system |
|  | Q5GRI5 | Wbm0097 | Cytoplasm | 167 |  | Fructose-bisphosphate aldolase (EC:4.1.2.13) | wbm00010 Glycolysis / Gluconeogenesis  wbm00030 Pentose phosphate pathway  wbm00051 Fructose and mannose metabolism  wbm00680 Methane metabolism  wbm01100 Metabolic pathways  wbm01110 Biosynthesis of secondary metabolites  wbm01120 Microbial metabolism in diverse environments  wbm01200 Carbon metabolism  wbm01230 Biosynthesis of amino acids |
|  | Q5GTH4 | Wbm0108 | Inner-membrane | 386 |  | 2-oxoglutarate dehydrogenase E2 component (dihydrolipoamide succinyltransferase) [EC:[2.3.1.61](http://www.genome.jp/dbget-bin/www_bget?ec:2.3.1.61)] | wbm00020 Citrate cycle (TCA cycle)  wbm00310 Lysine degradation  wbm01100 Metabolic pathways  wbm01110 Biosynthesis of secondary metabolites  wbm01120 Microbial metabolism in diverse environments  wbm01200 Carbon metabolism |
|  | Q5GTJ4 | Wbm0088 | Cytoplasm | 406 |  | Bifunctional UDP-N-acetylglucosamine pyrophosphorylase / Glucosamine-1-phosphate N-acetyltransferase [EC:[2.7.7.23](http://www.genome.jp/dbget-bin/www_bget?ec:2.7.7.23) [2.3.1.157](http://www.genome.jp/dbget-bin/www_bget?ec:2.3.1.157)] | wbm00520 Amino sugar and nucleotide sugar metabolism  wbm01100 Metabolic pathways  wbm01110 Biosynthesis of secondary metabolites |
|  | Q5GSI1 | Wbm0455 | Cytoplasm | 659 |  | Propionyl-CoA carboxylase alpha chain [EC:[6.4.1.3](http://www.genome.jp/dbget-bin/www_bget?ec:6.4.1.3)] | wbm00280 Valine, leucine and isoleucine degradation  wbm00630 Glyoxylate and dicarboxylate metabolism  wbm00640 Propanoate metabolism  wbm01100 Metabolic pathways  wbm01120 Microbial metabolism in diverse environments  wbm01200 Carbon metabolism |
|  | Q5GT10 | Wbm0275 | Cytoplasm | 242 |  | Glutamine synthetase [EC:[6.3.1.2](http://www.genome.jp/dbget-bin/www_bget?ec:6.3.1.2)] | wbm00250 Alanine, aspartate and glutamate metabolism  wbm00330 Arginine and proline metabolism  wbm00630 Glyoxylate and dicarboxylate metabolism  wbm00910 Nitrogen metabolism  wbm01100 Metabolic pathways  wbm01120 Microbial metabolism in diverse environments  wbm01230 Biosynthesis of amino acids  wbm02020 Two-component system |
|  | Q5GSM8 | Wbm0408 | Cytoplasm | 238 |  | Triosephosphate isomerase (TIM) [EC:[5.3.1.1](http://www.genome.jp/dbget-bin/www_bget?ec:5.3.1.1)] | wbm00010 Glycolysis / Gluconeogenesis  wbm00051 Fructose and mannose metabolism  wbm00562 Inositol phosphate metabolism  wbm01100 Metabolic pathways  wbm01110 Biosynthesis of secondary metabolites  wbm01120 Microbial metabolism in diverse environments  wbm01200 Carbon metabolism  wbm01230 Biosynthesis of amino acids |
|  | Q5GS11 | Wbm0625 | Inner-membrane | 177 | Yes | NADH-quinone oxidoreductase subunit J [EC:[1.6.5.3](http://www.genome.jp/dbget-bin/www_bget?ec:1.6.5.3)] | wbm00190 Oxidative phosphorylation |
|  | Q5GS12 | Wbm0624 | Inner-membrane | 102 |  | NADH-quinone oxidoreductase subunit K [EC:[1.6.5.3](http://www.genome.jp/dbget-bin/www_bget?ec:1.6.5.3)] | wbm00190 Oxidative phosphorylation |
|  | Q5GS13 | Wbm0623 | Inner-membrane | 618 | Yes | NADH-quinone oxidoreductase subunit L [EC:[1.6.5.3](http://www.genome.jp/dbget-bin/www_bget?ec:1.6.5.3)] | wbm00190 Oxidative phosphorylation |
|  | Q5GSF0 | Wbm0486 | Inner-membrane | 125 | Yes | Succinate dehydrogenase cytochrome b556 subunit | wbm00020 Citrate cycle (TCA cycle)  wbm00190 Oxidative phosphorylation  wbm01100 Metabolic pathways  wbm01110 Biosynthesis of secondary metabolites  wbm01120 Microbial metabolism in diverse environments  wbm01200 Carbon metabolism |
|  | Q5GSF1 | Wbm0485 | Inner-membrane | 121 | Yes | Succinate dehydrogenase membrane anchor subunit | wbm00020 Citrate cycle (TCA cycle)  wbm00190 Oxidative phosphorylation  wbm01100 Metabolic pathways  wbm01110 Biosynthesis of secondary metabolites  wbm01120 Microbial metabolism in diverse environments  wbm01200 Carbon metabolism |
|  | Q5GSX1 | Wbm0314 | Inner-membrane | 513 |  | F-type H+-transporting ATPase subunit alpha [EC:[3.6.3.14](http://www.genome.jp/dbget-bin/www_bget?ec:3.6.3.14)] | wbm00190 Oxidative phosphorylation |
|  | Q5GRU8 | Wbm0688 | Inner-membrane | 111 |  | F-type H+-transporting ATPase subunit epsilon | wbm00190 Oxidative phosphorylation |
|  | Q5GTA1 | Wbm0182 | Cytoplasm | 975 |  | Glutamate dehydrogenase [EC:[1.4.1.2](http://www.genome.jp/dbget-bin/www_bget?ec:1.4.1.2)] | wbm00250 Alanine, aspartate and glutamate metabolism  wbm00330 Arginine and proline metabolism  wbm00910 Nitrogen metabolism  wbm01100 Metabolic pathways |
|  | Q5GS22 | Wbm0614 | Cytoplasm | 326 |  | 3-oxoacyl-[acyl-carrier-protein] synthase III [EC:[2.3.1.180](http://www.genome.jp/dbget-bin/www_bget?ec:2.3.1.180)] | wbm00061 Fatty acid biosynthesis  wbm01100 Metabolic pathways  wbm01212 Fatty acid metabolism |
|  | Q5GRP7 | Wbm0739 | Inner-membrane | 422 |  | 3-oxoacyl-[acyl-carrier-protein] synthase II [EC:[2.3.1.179](http://www.genome.jp/dbget-bin/www_bget?ec:2.3.1.179)] | wbm00061 Fatty acid biosynthesis  wbm01100 Metabolic pathways  wbm01212 Fatty acid metabolism |
|  | Q5GTN0 | Wbm0052 | Cytoplasm | 146 |  | 3-hydroxyacyl-[acyl-carrier-protein] dehydratase [EC:[4.2.1.59](http://www.genome.jp/dbget-bin/www_bget?ec:4.2.1.59)] | wbm00061 Fatty acid biosynthesis  wbm00780 Biotin metabolism  wbm01100 Metabolic pathways  wbm01212 Fatty acid metabolism |
|  | Q5GRN3 | Wbm0753 | Cytoplasm | 346 |  | Enoyl-[acyl-carrier protein] reductase II [EC:1.3.1.-] | wbm00061 Fatty acid biosynthesis  wbm01100 Metabolic pathways  wbm01212 Fatty acid metabolism |
|  | Q5GSL3 | Wbm0423 | Inner-membrane | 191 | Yes | Glycerol-3-phosphate acyltransferase PlsY [EC:[2.3.1.15](http://www.genome.jp/dbget-bin/www_bget?ec:2.3.1.15)] | wbm00561 Glycerolipid metabolism  wbm00564 Glycerophospholipid metabolism  wbm01100 Metabolic pathways |
|  | Q5GSL1 | Wbm0425 | Inner-membrane | 256 | Yes | CDP-diacylglycerol---serine O-phosphatidyltransferase [EC:[2.7.8.8](http://www.genome.jp/dbget-bin/www_bget?ec:2.7.8.8)] | wbm00260 Glycine, serine and threonine metabolism  wbm00564 Glycerophospholipid metabolism  wbm01100 Metabolic pathways |
|  | Q5GSL0 | Wbm0426 | Cytoplasm | 230 | Yes | Phosphatidylserine decarboxylase [EC:[4.1.1.65](http://www.genome.jp/dbget-bin/www_bget?ec:4.1.1.65)] | wbm00564 Glycerophospholipid metabolism |
|  | Q5GTP1 | Wbm0041 | Cytoplasm | 357 |  | 5-(carboxyamino)imidazole ribonucleotide synthase [EC:[6.3.4.18](http://www.genome.jp/dbget-bin/www_bget?ec:6.3.4.18)] | wbm00230 Purine metabolism  wbm01100 Metabolic pathways  wbm01110 Biosynthesis of secondary metabolites |
|  | Q5GSN9 | Wbm0397 | Cytoplasm | 167 |  | 5-(carboxyamino)imidazole ribonucleotide mutase [EC:[5.4.99.18](http://www.genome.jp/dbget-bin/www_bget?ec:5.4.99.18)] | wbm00230 Purine metabolism  wbm01100 Metabolic pathways  wbm01110 Biosynthesis of secondary metabolites |
|  | Q5GSW7 | Wbm0318 | Cytoplasm | 355 |  | DNA-directed RNA polymerase subunit alpha [EC:[2.7.7.6](http://www.genome.jp/dbget-bin/www_bget?ec:2.7.7.6)] | wbm00230 Purine metabolism  wbm00240 Pyrimidine metabolism  wbm01100 Metabolic pathways  wbm03020 RNA polymerase |
|  | Q5GS87 | Wbm0549 | Periplasm | 250 |  | 5'-nucleotidase [EC:[3.1.3.5](http://www.genome.jp/dbget-bin/www_bget?ec:3.1.3.5)] | wbm00230 Purine metabolism  wbm00240 Pyrimidine metabolism  wbm00760 Nicotinate and nicotinamide metabolism  wbm01100 Metabolic pathways  wbm01110 Biosynthesis of secondary metabolites |
|  | Q5GTA7 | Wbm0176 | Cytoplasm | 336 |  | DNA polymerase III subunit delta [EC:[2.7.7.7](http://www.genome.jp/dbget-bin/www_bget?ec:2.7.7.7)] | wbm00230 Purine metabolism  wbm00240 Pyrimidine metabolism  wbm01100 Metabolic pathways  wbm03030 DNA replication  wbm03430 Mismatch repair  wbm03440 Homologous recombination |
|  | Q5GTQ7 | Wbm0025 | Cytoplasm | 399 |  | dGTPase [EC:[3.1.5.1](http://www.genome.jp/dbget-bin/www_bget?ec:3.1.5.1)] | Purine Metabolism |
|  | Q5GRJ9 | Wbm0787 | Cytoplasm | 255 |  | Orotidine-5'-phosphate decarboxylase [EC:[4.1.1.23](http://www.genome.jp/dbget-bin/www_bget?ec:4.1.1.23)] | wbm00240 Pyrimidine metabolism  wbm01100 Metabolic pathways |
|  | Q5GSZ3 | Wbm0292 | Extra-Cellular | 185 |  | Deoxycytidine triphosphate deaminase (EC:3.5.4.13) | wbm00240 Pyrimidine metabolism  wbm01100 Metabolic pathways |
|  | Q5GRI0 | Wbm0806 | Cytoplasm | 246 |  | Uridylate kinase [EC:[2.7.4.22](http://www.genome.jp/dbget-bin/www_bget?ec:2.7.4.22)] | wbm00240 Pyrimidine metabolism  wbm01100 Metabolic pathways |
|  | Q5GSJ5 | Wbm0441 | Cytoplasm | 401 |  | Aspartate kinase [EC:[2.7.2.4](http://www.genome.jp/dbget-bin/www_bget?ec:2.7.2.4)] | wbm00260 Glycine, serine and threonine metabolism  wbm00270 Cysteine and methionine metabolism  wbm00300 Lysine biosynthesis  wbm01100 Metabolic pathways  wbm01110 Biosynthesis of secondary metabolites  wbm01120 Microbial metabolism in diverse environments  wbm01210 2-Oxocarboxylic acid metabolism  wbm01230 Biosynthesis of amino acids |
|  | Q5GTP0 | Wbm0042 | Cytoplasm | 347 |  | Aspartate-semialdehyde dehydrogenase [EC:[1.2.1.11](http://www.genome.jp/dbget-bin/www_bget?ec:1.2.1.11)] | wbm00260 Glycine, serine and threonine metabolism  wbm00270 Cysteine and methionine metabolism  wbm00300 Lysine biosynthesis  wbm01100 Metabolic pathways  wbm01110 Biosynthesis of secondary metabolites  wbm01120 Microbial metabolism in diverse environments  wbm01210 2-Oxocarboxylic acid metabolism  wbm01230 Biosynthesis of amino acids |
|  | Q5GTA6 | Wbm0177 | Cytoplasm | 264 |  | 4-hydroxy-tetrahydrodipicolinate reductase [EC:[1.17.1.8](http://www.genome.jp/dbget-bin/www_bget?ec:1.17.1.8)] | wbm00300 Lysine biosynthesis  wbm01100 Metabolic pathways  wbm01110 Biosynthesis of secondary metabolites  wbm01120 Microbial metabolism in diverse environments  wbm01230 Biosynthesis of amino acids |
|  | Q5GSI7 | Wbm0449 | Cytoplasm | 273 |  | 2,3,4,5-tetrahydropyridine-2-carboxylate N-succinyltransferase [EC:[2.3.1.117](http://www.genome.jp/dbget-bin/www_bget?ec:2.3.1.117)] | wbm00300 Lysine biosynthesis  wbm01100 Metabolic pathways  wbm01120 Microbial metabolism in diverse environments  wbm01230 Biosynthesis of amino acids |
|  | Q5GSB8 | Wbm0518 | Cytoplasm | 259 |  | Diaminopimelate epimerase [EC:[5.1.1.7](http://www.genome.jp/dbget-bin/www_bget?ec:5.1.1.7)] | wbm00300 Lysine biosynthesis  wbm01100 Metabolic pathways  wbm01110 Biosynthesis of secondary metabolites  wbm01120 Microbial metabolism in diverse environments  wbm01230 Biosynthesis of amino acids |
|  | Q5GS70 | Wbm0566 | Cytoplasm | 318 | Yes | Thioredoxin reductase (NADPH) [EC:[1.8.1.9](http://www.genome.jp/dbget-bin/www_bget?ec:1.8.1.9)] | wbm00240 Pyrimidine metabolism  wbm00450 Selenocompound metabolism |
|  | Q5GS80 | Wbm0556 | Cytoplasm | 305 |  | Glutathione synthase [EC:[6.3.2.3](http://www.genome.jp/dbget-bin/www_bget?ec:6.3.2.3)] | wbm00480 Glutathione metabolism  wbm01100 Metabolic pathways |
|  | Q5GT07 | Wbm0278 | Extra-Cellular | 360 |  | GTP cyclohydrolase II [EC:[3.5.4.25](http://www.genome.jp/dbget-bin/www_bget?ec:3.5.4.25)] | wbm00740 Riboflavin metabolism  wbm01100 Metabolic pathways |
|  | Q5GTQ6 | Wbm0026 | Cytoplasm | 378 |  | Diaminohydroxyphosphoribosylaminopyrimidine deaminase / 5-amino-6-(5-phosphoribosylamino)uracil reductase [EC:[3.5.4.26](http://www.genome.jp/dbget-bin/www_bget?ec:3.5.4.26) [1.1.1.193](http://www.genome.jp/dbget-bin/www_bget?ec:1.1.1.193)] | wbm00740 Riboflavin metabolism  wbm01100 Metabolic pathways |
|  | Q5GSX3 | Wbm0312 | Periplasm | 217 |  | 3,4-dihydroxy 2-butanone 4-phosphate synthase [EC:[4.1.99.12](http://www.genome.jp/dbget-bin/www_bget?ec:4.1.99.12)] | wbm00740 Riboflavin metabolism  wbm01100 Metabolic pathways |
|  | Q5GT94 | Wbm0189 | Cytoplasm | 142 |  | 6,7-dimethyl-8-ribityllumazine synthase [EC:[2.5.1.78](http://www.genome.jp/dbget-bin/www_bget?ec:2.5.1.78)] | wbm00740 Riboflavin metabolism  wbm01100 Metabolic pathways |
|  | Q5GTJ9 | Wbm0083 | Cytoplasm | 198 |  | Riboflavin synthase [EC:[2.5.1.9](http://www.genome.jp/dbget-bin/www_bget?ec:2.5.1.9)] | wbm00740 Riboflavin metabolism  wbm01100 Metabolic pathways |
|  | Q5GSN6 | Wbm0400 | Cytoplasm | 264 |  | Inorganic polyphosphate/ATP-NAD kinase (EC:2.7.1.23) | wbm00760 Nicotinate and nicotinamide metabolism  wbm01100 Metabolic pathways |
|  | Q5GTK4 | Wbm0078 | Cytoplasm | 125 |  | Holo-[acyl-carrier protein] synthase [EC:[2.7.8.7](http://www.genome.jp/dbget-bin/www_bget?ec:2.7.8.7)] | wbm00770 Pantothenate and CoA biosynthesis |
|  | Q5GRQ8 | Wbm0728 | Cytoplasm | 235 |  | Uroporphyrinogen-III synthase [EC:[4.2.1.75](http://www.genome.jp/dbget-bin/www_bget?ec:4.2.1.75)] | wbm00860 Porphyrin and chlorophyll metabolism  wbm01100 Metabolic pathways  wbm01110 Biosynthesis of secondary metabolites |
|  | Q5GRR7 | Wbm0719 | Cytoplasm | 340 |  | Ferrochelatase [EC:[4.99.1.1](http://www.genome.jp/dbget-bin/www_bget?ec:4.99.1.1)] | wbm00860 Porphyrin and chlorophyll metabolism  wbm01100 Metabolic pathways  wbm01110 Biosynthesis of secondary metabolites |
|  | Q5GTL0 | Wbm0072 | Cytoplasm | 187 |  | 3-octaprenyl-4-hydroxybenzoate carboxy-lyase UbiX [EC:4.1.1.-] | wbm00130 Ubiquinone and other terpenoid-quinone biosynthesis  wbm01100 Metabolic pathways  wbm01110 Biosynthesis of secondary metabolites |
|  | Q5GTA4 | Wbm0179 | Cytoplasm | 389 |  | 1-deoxy-D-xylulose-5-phosphate reductoisomerase [EC:[1.1.1.267](http://www.genome.jp/dbget-bin/www_bget?ec:1.1.1.267)] | wbm00900 Terpenoid backbone biosynthesis  wbm01100 Metabolic pathways  wbm01110 Biosynthesis of secondary metabolites |
|  | Q5GTB0 | Wbm0173 | Cytoplasm | 288 |  | 4-diphosphocytidyl-2-C-methyl-D-erythritol kinase [EC:[2.7.1.148](http://www.genome.jp/dbget-bin/www_bget?ec:2.7.1.148)] | wbm00900 Terpenoid backbone biosynthesis  wbm01100 Metabolic pathways  wbm01110 Biosynthesis of secondary metabolites |
|  | Q5GRK4 | Wbm0782 | Cytoplasm | 429 |  | E)-4-hydroxy-3-methylbut-2-enyl-diphosphate synthase [EC:[1.17.7.1](http://www.genome.jp/dbget-bin/www_bget?ec:1.17.7.1)] | wbm00900 Terpenoid backbone biosynthesis  wbm01100 Metabolic pathways  wbm01110 Biosynthesis of secondary metabolites |
|  | Q5GTN6 | Wbm0046 | Periplasm | 309 |  | 4-hydroxy-3-methylbut-2-enyl diphosphate reductase (EC:1.17.1.2) | wbm00900 Terpenoid backbone biosynthesis  wbm01100 Metabolic pathways  wbm01110 Biosynthesis of secondary metabolites |
|  | Q5GSP9 | Wbm0387 | Cytoplasm | 142 |  | DNA-directed RNA polymerase subunit omega [EC:[2.7.7.6](http://www.genome.jp/dbget-bin/www_bget?ec:2.7.7.6)] | wbm00230 Purine metabolism  wbm00240 Pyrimidine metabolism  wbm01100 Metabolic pathways  wbm03020 RNA polymerase |
|  | Q5GSU3 | Wbm0342 | Cytoplasm | 121 |  | Small subunit ribosomal protein S10 | wbm03010 Ribosome |
|  | Q5GSV0 | Wbm0335 | Cytoplasm | 206 |  | Small subunit ribosomal protein S3 | wbm03010 Ribosome |
|  | Q5GSV3 | Wbm0332 |  |  |  | Small subunit ribosomal protein S17 | wbm03010 Ribosome |
|  | Q5GSV8 | Wbm0327 | Cytoplasm | 131 |  | Small subunit ribosomal protein S8 | wbm03010 Ribosome |
|  | Q5GSV9 | Wbm0326 | Cytoplasm | 181 |  | Large subunit ribosomal protein L6 | wbm03010 Ribosome |
|  | Q5GSW0 | Wbm0325 | Cytoplasm | 123 |  | Large subunit ribosomal protein L18 | wbm03010 Ribosome |
|  | Q5GS04 | Wbm0632 |  |  |  | Large subunit ribosomal protein L36 | wbm03010 Ribosome |
|  | Q5GRY7 | Wbm0649 | Cytoplasm | 172 |  | Large subunit ribosomal protein L10 | wbm03010 Ribosome |
|  | Q5GTR2 | Wbm0020 | Cytoplasm | 121 |  | Small subunit ribosomal protein S15 | wbm03010 Ribosome |
|  | Q5GRX8 | Wbm0658 |  |  |  | Large subunit ribosomal protein L35 | wbm03010 Ribosome |
|  | Q5GRX9 | Wbm0657 |  |  |  | Large subunit ribosomal protein L20 | wbm03010 Ribosome |
|  | Q5GRU5 | Wbm0691 |  |  |  | Large subunit ribosomal protein L34 | wbm03010 Ribosome |
|  | Q5GSN5 | Wbm0401 |  |  |  | Large subunit ribosomal protein L31 | wbm03010 Ribosome |
|  | Q5GSD4 | Wbm0502 | Cytoplasm | 186 |  | Large subunit ribosomal protein L9 | wbm03010 Ribosome |
|  | Q5GSD5 | Wbm0501 |  |  |  | Small subunit ribosomal protein S18 | wbm03010 Ribosome |
|  | Q5GSD6 | Wbm0500 | Cytoplasm | 260 |  | Small subunit ribosomal protein S6 | wbm03010 Ribosome |
|  | Q5GRT6 | Wbm0700 |  |  |  | Large subunit ribosomal protein L27 | wbm03010 Ribosome |
|  | Q5GRQ2 | Wbm0734 |  |  |  | Small subunit ribosomal protein S20 | wbm03010 Ribosome |
|  | Q5GTI0 | Wbm0102 | Cytoplasm | 117 |  | Aspartyl-tRNA(Asn)/glutamyl-tRNA(Gln) Amidotransferase subunit C [EC:[6.3.5.6](http://www.genome.jp/dbget-bin/www_bget?ec:6.3.5.6) [6.3.5.7](http://www.genome.jp/dbget-bin/www_bget?ec:6.3.5.7)] | wbm00970 Aminoacyl-tRNA biosynthesis  wbm01100 Metabolic pathways |
|  | Q5GSR6 | Wbm0370 | Cytoplasm | 279 |  | Glycyl-tRNA synthetase alpha chain [EC:[6.1.1.14](http://www.genome.jp/dbget-bin/www_bget?ec:6.1.1.14)] | wbm00970 Aminoacyl-tRNA biosynthesis |
|  | Q5GRI7 | Wbm0799 | Cytoplasm | 479 |  | Lysyl-tRNA synthetase, class I [EC:[6.1.1.6](http://www.genome.jp/dbget-bin/www_bget?ec:6.1.1.6)] | wbm00970 Aminoacyl-tRNA biosynthesis |
|  | Q5GRM6 | Wbm0760 | Inner-Membrane | 205 |  | Signal peptidase I [EC:[3.4.21.89](http://www.genome.jp/dbget-bin/www_bget?ec:3.4.21.89)] | wbm03060 Protein export |
|  | Q5GSL9 | Wbm0417 | Inner-Membrane | 154 | Yes | Signal peptidase II [EC:[3.4.23.36](http://www.genome.jp/dbget-bin/www_bget?ec:3.4.23.36)] | wbm03060 Protein export |
|  | Q5GSE0 | Wbm0496 | Cytoplasm | 590 |  | Ribonuclease E [EC:[3.1.26.12](http://www.genome.jp/dbget-bin/www_bget?ec:3.1.26.12)] | wbm03018 RNA degradation |
|  | Q5GT39 | Wbm0246 | Extracellular | 161 |  | Putative (di)nucleoside polyphosphate hydrolase [EC:3.6.1.-] | wbm03018 RNA degradation |
|  | Q5GS93 | Wbm0543 | Cytoplasm | 231 |  | DNA polymerase III subunit epsilon [EC:[2.7.7.7](http://www.genome.jp/dbget-bin/www_bget?ec:2.7.7.7)] | wbm00230 Purine metabolism  wbm00240 Pyrimidine metabolism  wbm01100 Metabolic pathways  wbm03030 DNA replication  wbm03430 Mismatch repair  wbm03440 Homologous recombination |
|  | Q5GSD7 | Wbm0499 | Cytoplasm | 975 |  | DNA polymerase III subunit alpha [EC:[2.7.7.7](http://www.genome.jp/dbget-bin/www_bget?ec:2.7.7.7)] | wbm00230 Purine metabolism  wbm00240 Pyrimidine metabolism  wbm01100 Metabolic pathways  wbm03030 DNA replication  wbm03430 Mismatch repair  wbm03440 Homologous recombination |
|  | Q5GSK2 | Wbm0434 | Cytoplasm | 494 |  | DNA polymerase III subunit gamma/tau [EC:[2.7.7.7](http://www.genome.jp/dbget-bin/www_bget?ec:2.7.7.7)] | wbm00230 Purine metabolism  wbm00240 Pyrimidine metabolism  wbm01100 Metabolic pathways  wbm03030 DNA replication  wbm03430 Mismatch repair  wbm03440 Homologous recombination |
|  | Q5GSK7 | Wbm0429 | Cytoplasm | 393 |  | DNA polymerase III subunit beta [EC:[2.7.7.7](http://www.genome.jp/dbget-bin/www_bget?ec:2.7.7.7)] | wbm00230 Purine metabolism  wbm00240 Pyrimidine metabolism  wbm01100 Metabolic pathways  wbm03030 DNA replication  wbm03430 Mismatch repair  wbm03440 Homologous recombination |
|  | Q5GSY6 | Wbm0299 | Nucleoid | 483 |  | Replicative DNA helicase [EC:[3.6.4.12](http://www.genome.jp/dbget-bin/www_bget?ec:3.6.4.12)] | wbm03030 DNA replication |
|  | Q5GT89 | Wbm0194 | Cytoplasm | 586 |  | DNA primase [EC:2.7.7.-] | wbm03030 DNA replication |
|  | Q5GS47 | Wbm0589 | Cytoplasm | 271 |  | Formamidopyrimidine-DNA glycosylase [EC:[3.2.2.23](http://www.genome.jp/dbget-bin/www_bget?ec:3.2.2.23)[4.2.99.18](http://www.genome.jp/dbget-bin/www_bget?ec:4.2.99.18)] | wbm03410 Base excision repair |
|  | Q5GTF9 | Wbm0124 | Cytoplasm | 579 |  | Single-stranded-DNA-specific exonuclease [EC:3.1.-.-] | wbm03410 Base excision repair  wbm03430 Mismatch repair  wbm03440 Homologous recombination |
|  | Q5GSR4 | Wbm0372 | Cytoplasm | 606 |  | Excinuclease ABC subunit C | wbm03420 Nucleotide excision repair |
|  | Q5GS08 | Wbm0628 | Cytoplasm | 638 |  | DNA helicase II / ATP-dependent DNA helicase PcrA [EC:[3.6.4.12](http://www.genome.jp/dbget-bin/www_bget?ec:3.6.4.12)] | wbm03420 Nucleotide excision repair  wbm03430 Mismatch repair |
|  | Q5GT34 | Wbm0251 | Cytoplasm | 196 |  | Holliday junction DNA helicase RuvA [EC:[3.6.4.12](http://www.genome.jp/dbget-bin/www_bget?ec:3.6.4.12)] | wbm03440 Homologous recombination |
|  | Q5GRT9 | Wbm0697 | Cytoplasm | 162 |  | Crossover junction endodeoxyribonuclease RuvC [EC:[3.1.22.4](http://www.genome.jp/dbget-bin/www_bget?ec:3.1.22.4)] | wbm03440 Homologous recombination |
|  | Q5GRP1 | Wbm0745 | Cytoplasm | 786 |  | Primosomal protein N' (replication factor Y) (superfamily II helicase) [EC:3.6.4.-] | wbm03440 Homologous recombination wbm03440 Homologous recombination |
|  | Q5GS61 | Wbm0575 | Periplasm | 411 |  | Outer membrane channel protein | wbm00312 beta-Lactam resistance  wbm03070 Bacterial secretion system |

**Supplementary Table 3:** Potential drug targets of *Wbm.*

| **Sl. No.** | **UniProt ID** | **KEGG entry** | **Sub-cellular Localization**  (Gneg-mPLoc) | **Seq Length** | **Protein name** | **Pathways name and ID** |
| --- | --- | --- | --- | --- | --- | --- |
| 1. **1** | Q5GTC5 | *Wbm*0158 | Cytoplasm | 308 | Fructose 1,6-bisphosphatase II (EC:3.1.3.11) | *Wbm*00010 Glycolysis / Gluconeogenesis  *Wbm*00030 Pentose phosphate pathway  *Wbm*00051 Fructose and mannose metabolism  *Wbm*00680 Methane metabolism  *Wbm*01100 Metabolic pathways  *Wbm*01110 Biosynthesis of secondary metabolites  *Wbm*01120 Microbial metabolism in diverse environments  *Wbm*01200 Carbon metabolism |
|  | Q5GSN3 | *Wbm*0403 | Cytoplasm | 501 | 2,3-bisphosphoglycerate-independent phosphoglycerate mutase [EC:[5.4.2.12](http://www.genome.jp/dbget-bin/www_bget?ec:5.4.2.12)] | *Wbm*00010 Glycolysis / Gluconeogenesis  *Wbm*00260 Glycine, serine and threonine metabolism  *Wbm*00680 Methane metabolism  *Wbm*01100 Metabolic pathways  *Wbm*01110 Biosynthesis of secondary metabolites  *Wbm*01120 Microbial metabolism in diverse environments  *Wbm*01200 Carbon metabolism  *Wbm*01230 Biosynthesis of amino acids |
|  | Q5GRP6 | *Wbm*0740 | Inner-Membrane | 425 | UDP-N-acetylglucosamine 1-carboxyvinyltransferase [EC:[2.5.1.7](http://www.genome.jp/dbget-bin/www_bget?ec:2.5.1.7)] | *Wbm*00520 Amino sugar and nucleotide sugar metabolism  *Wbm*00550 Peptidoglycan biosynthesis  *Wbm*01100 Metabolic pathways |
|  | Q5GRK8 | *Wbm*0778 | Inner-Membrane | 295 | UDP-N-acetylmuramate dehydrogenase [EC:[1.3.1.98](http://www.genome.jp/dbget-bin/www_bget?ec:1.3.1.98)] | *Wbm*00520 Amino sugar and nucleotide sugar metabolism  *Wbm*00550 Peptidoglycan biosynthesis  *Wbm*01100 Metabolic pathways |
|  | Q5GTG5 | *Wbm*0118 | Cytoplasm | 536 | UDP-N-acetylmuramate--alanine ligase [EC:[6.3.2.8](http://www.genome.jp/dbget-bin/www_bget?ec:6.3.2.8)] | *Wbm*00471 D-Glutamine and D-glutamate metabolism  *Wbm*00550 Peptidoglycan biosynthesis  *Wbm*01100 Metabolic pathways |
|  | Q5GSC8 | *Wbm*0508 | Cytoplasm | 498 | UDP-N-acetylmuramoylalanine--D-glutamate ligase [EC:[6.3.2.9](http://www.genome.jp/dbget-bin/www_bget?ec:6.3.2.9)] | *Wbm*00471 D-Glutamine and D-glutamate metabolism  *Wbm*00550 Peptidoglycan biosynthesis  *Wbm*01100 Metabolic pathways |
|  | Q5GSE4 | *Wbm*0492 | Cytoplasm | 496 | UDP-N-acetylmuramoyl-L-alanyl-D-glutamate--2,6-diaminopimelate ligase [EC:[6.3.2.13](http://www.genome.jp/dbget-bin/www_bget?ec:6.3.2.13)] | *Wbm*00300 Lysine biosynthesis  *Wbm*00550 Peptidoglycan biosynthesis |
|  | Q5GT47 | *Wbm*0238 | Cytoplasm | 455 | UDP-N-acetylmuramoyl-tripeptide--D-alanyl-D-alanine ligase [EC:[6.3.2.10](http://www.genome.jp/dbget-bin/www_bget?ec:6.3.2.10)] | *Wbm*00300 Lysine biosynthesis  *Wbm*00550 Peptidoglycan biosynthesis  *Wbm*01100 Metabolic pathways |
|  | Q5GSZ5 | *Wbm*0290 | Cytoplasm | 370 | D-alanyl-D-alanine carboxypeptidase (penicillin-binding protein 5/6) [EC:[3.4.16.4](http://www.genome.jp/dbget-bin/www_bget?ec:3.4.16.4)] | *Wbm*00550 Peptidoglycan biosynthesis  *Wbm*01100 Metabolic pathways |
|  | Q5GT19 | *Wbm*0266 | Inner-membrane | 867 | Preprotein translocase subunit SecA | *Wbm*03060 Protein export  *Wbm*03070 Bacterial secretion system |
|  | Q5GS95 | *Wbm*0541 | Cytoplasm | 167 | Preprotein translocase subunit SecB | *Wbm*03060 Protein export  *Wbm*03070 Bacterial secretion system |
|  | Q5GT03 | *Wbm*0282 | Inner-membrane | 335 | Type IV secretion system protein VirB11 | *Wbm*03070 Bacterial secretion system |
|  | Q5GT09 | *Wbm*0276 | Cytoplasm | 460 | Chromosomal replication initiator protein | *Wbm*02020 Two-component system |
|  | Q5GT10 | *Wbm*0275 | Cytoplasm | 242 | Glutamine synthetase [EC:[6.3.1.2](http://www.genome.jp/dbget-bin/www_bget?ec:6.3.1.2)] | *Wbm*00250 Alanine, aspartate and glutamate metabolism  *Wbm*00330 Arginine and proline metabolism  *Wbm*00630 Glyoxylate and dicarboxylate metabolism  *Wbm*00910 Nitrogen metabolism  *Wbm*01100 Metabolic pathways  *Wbm*01120 Microbial metabolism in diverse environments  *Wbm*01230 Biosynthesis of amino acids  *Wbm*02020 Two-component system |
|  | Q5GT99 | *Wbm*0184 | Cytoplasm | 448 | Two-component system, cell cycle response regulator | *Wbm*02020 Two-component system |
|  | Q5GRI5 | *Wbm*0097 | Cytoplasm | 167 | Fructose-bisphosphate aldolase (EC:4.1.2.13) | *Wbm*00010 Glycolysis / Gluconeogenesis  *Wbm*00030 Pentose phosphate pathway  *Wbm*00051 Fructose and mannose metabolism  *Wbm*00680 Methane metabolism  *Wbm*01100 Metabolic pathways  *Wbm*01110 Biosynthesis of secondary metabolites  *Wbm*01120 Microbial metabolism in diverse environments  *Wbm*01200 Carbon metabolism  *Wbm*01230 Biosynthesis of amino acids |
|  | Q5GTH4 | *Wbm*0108 | Inner-membrane | 386 | 2-oxoglutarate dehydrogenase E2 component (dihydrolipoamide succinyltransferase) [EC:[2.3.1.61](http://www.genome.jp/dbget-bin/www_bget?ec:2.3.1.61)] | *Wbm*00020 Citrate cycle (TCA cycle)  *Wbm*00310 Lysine degradation  *Wbm*01100 Metabolic pathways  *Wbm*01110 Biosynthesis of secondary metabolites  *Wbm*01120 Microbial metabolism in diverse environments  *Wbm*01200 Carbon metabolism |
|  | Q5GTJ4 | *Wbm*0088 | Cytoplasm | 406 | Bifunctional UDP-N-acetylglucosamine pyrophosphorylase / Glucosamine-1-phosphate N-acetyltransferase [EC:[2.7.7.23](http://www.genome.jp/dbget-bin/www_bget?ec:2.7.7.23) [2.3.1.157](http://www.genome.jp/dbget-bin/www_bget?ec:2.3.1.157)] | *Wbm*00520 Amino sugar and nucleotide sugar metabolism  *Wbm*01100 Metabolic pathways  *Wbm*01110 Biosynthesis of secondary metabolites |
|  | Q5GSI1 | *Wbm*0455 | Cytoplasm | 659 | Propionyl-CoA carboxylase alpha chain [EC:[6.4.1.3](http://www.genome.jp/dbget-bin/www_bget?ec:6.4.1.3)] | *Wbm*00280 Valine, leucine and isoleucine degradation  *Wbm*00630 Glyoxylate and dicarboxylate metabolism  *Wbm*00640 Propanoate metabolism  *Wbm*01100 Metabolic pathways  *Wbm*01120 Microbial metabolism in diverse environments  *Wbm*01200 Carbon metabolism |
|  | Q5GSM8 | *Wbm*0408 | Cytoplasm | 238 | Triosephosphate isomerase (TIM) [EC:[5.3.1.1](http://www.genome.jp/dbget-bin/www_bget?ec:5.3.1.1)] | *Wbm*00010 Glycolysis / Gluconeogenesis  *Wbm*00051 Fructose and mannose metabolism  *Wbm*00562 Inositol phosphate metabolism  *Wbm*01100 Metabolic pathways  *Wbm*01110 Biosynthesis of secondary metabolites  *Wbm*01120 Microbial metabolism in diverse environments  *Wbm*01200 Carbon metabolism  *Wbm*01230 Biosynthesis of amino acids |
|  | Q5GSX1 | *Wbm*0314 | Inner-membrane | 513 | F-type H+-transporting ATPase subunit alpha [EC:[3.6.3.14](http://www.genome.jp/dbget-bin/www_bget?ec:3.6.3.14)] | *Wbm*00190 Oxidative phosphorylation |
|  | Q5GRU8 | *Wbm*0688 | Inner-membrane | 111 | F-type H+-transporting ATPase subunit epsilon | *Wbm*00190 Oxidative phosphorylation |
|  | Q5GTA1 | *Wbm*0182 | Cytoplasm | 975 | Glutamate dehydrogenase [EC:[1.4.1.2](http://www.genome.jp/dbget-bin/www_bget?ec:1.4.1.2)] | *Wbm*00250 Alanine, aspartate and glutamate metabolism  *Wbm*00330 Arginine and proline metabolism  *Wbm*00910 Nitrogen metabolism  *Wbm*01100 Metabolic pathways |
|  | Q5GRP7 | *Wbm*0739 | Inner-membrane | 422 | 3-oxoacyl-[acyl-carrier-protein] synthase II [EC:[2.3.1.179](http://www.genome.jp/dbget-bin/www_bget?ec:2.3.1.179)] | *Wbm*00061 Fatty acid biosynthesis  *Wbm*01100 Metabolic pathways  *Wbm*01212 Fatty acid metabolism |
|  | Q5GTN0 | *Wbm*0052 | Cytoplasm | 146 | 3-hydroxyacyl-[acyl-carrier-protein] dehydratase [EC:[4.2.1.59](http://www.genome.jp/dbget-bin/www_bget?ec:4.2.1.59)] | *Wbm*00061 Fatty acid biosynthesis  *Wbm*00780 Biotin metabolism  *Wbm*01100 Metabolic pathways  *Wbm*01212 Fatty acid metabolism |
|  | Q5GRN3 | *Wbm*0753 | Cytoplasm | 346 | Enoyl-[acyl-carrier protein] reductase II [EC:1.3.1.-] | *Wbm*00061 Fatty acid biosynthesis  *Wbm*01100 Metabolic pathways  *Wbm*01212 Fatty acid metabolism |
|  | Q5GTP1 | *Wbm*0041 | Cytoplasm | 357 | 5-(carboxyamino)imidazole ribonucleotide synthase [EC:[6.3.4.18](http://www.genome.jp/dbget-bin/www_bget?ec:6.3.4.18)] | *Wbm*00230 Purine metabolism  *Wbm*01100 Metabolic pathways  *Wbm*01110 Biosynthesis of secondary metabolites |
|  | Q5GSN9 | *Wbm*0397 | Cytoplasm | 167 | 5-(carboxyamino)imidazole ribonucleotide mutase [EC:[5.4.99.18](http://www.genome.jp/dbget-bin/www_bget?ec:5.4.99.18)] | *Wbm*00230 Purine metabolism  *Wbm*01100 Metabolic pathways  *Wbm*01110 Biosynthesis of secondary metabolites |
|  | Q5GSW7 | *Wbm*0318 | Cytoplasm | 355 | DNA-directed RNA polymerase subunit alpha [EC:[2.7.7.6](http://www.genome.jp/dbget-bin/www_bget?ec:2.7.7.6)] | *Wbm*00230 Purine metabolism  *Wbm*00240 Pyrimidine metabolism  *Wbm*01100 Metabolic pathways  *Wbm*03020 RNA polymerase |
|  | Q5GS87 | *Wbm*0549 | Periplasm | 250 | 5'-nucleotidase [EC:[3.1.3.5](http://www.genome.jp/dbget-bin/www_bget?ec:3.1.3.5)] | *Wbm*00230 Purine metabolism  *Wbm*00240 Pyrimidine metabolism  *Wbm*00760 Nicotinate and nicotinamide metabolism  *Wbm*01100 Metabolic pathways  *Wbm*01110 Biosynthesis of secondary metabolites |
|  | Q5GTA7 | *Wbm*0176 | Cytoplasm | 336 | DNA polymerase III subunit delta [EC:[2.7.7.7](http://www.genome.jp/dbget-bin/www_bget?ec:2.7.7.7)] | *Wbm*00230 Purine metabolism  *Wbm*00240 Pyrimidine metabolism  *Wbm*01100 Metabolic pathways  *Wbm*03030 DNA replication  *Wbm*03430 Mismatch repair  *Wbm*03440 Homologous recombination |
|  | Q5GTQ7 | *Wbm*0025 | Cytoplasm | 399 | dGTPase [EC:[3.1.5.1](http://www.genome.jp/dbget-bin/www_bget?ec:3.1.5.1)] | Purine Metabolism |
|  | Q5GRJ9 | *Wbm*0787 | Cytoplasm | 225 | Orotidine-5'-phosphate decarboxylase [EC:[4.1.1.23](http://www.genome.jp/dbget-bin/www_bget?ec:4.1.1.23)] | *Wbm*00240 Pyrimidine metabolism  *Wbm*01100 Metabolic pathways |
|  | Q5GRI0 | *Wbm*0806 | Cytoplasm | 246 | Uridylate kinase [EC:[2.7.4.22](http://www.genome.jp/dbget-bin/www_bget?ec:2.7.4.22)] | *Wbm*00240 Pyrimidine metabolism  *Wbm*01100 Metabolic pathways |
|  | Q5GSJ5 | *Wbm*0441 | Cytoplasm | 401 | Aspartate kinase [EC:[2.7.2.4](http://www.genome.jp/dbget-bin/www_bget?ec:2.7.2.4)] | *Wbm*00260 Glycine, serine and threonine metabolism  *Wbm*00270 Cysteine and methionine metabolism  *Wbm*00300 Lysine biosynthesis  *Wbm*01100 Metabolic pathways  *Wbm*01110 Biosynthesis of secondary metabolites  *Wbm*01120 Microbial metabolism in diverse environments  *Wbm*01210 2-Oxocarboxylic acid metabolism  *Wbm*01230 Biosynthesis of amino acids |
|  | Q5GTP0 | *Wbm*0042 | Cytoplasm | 347 | Aspartate-semialdehyde dehydrogenase [EC:[1.2.1.11](http://www.genome.jp/dbget-bin/www_bget?ec:1.2.1.11)] | *Wbm*00260 Glycine, serine and threonine metabolism  *Wbm*00270 Cysteine and methionine metabolism  *Wbm*00300 Lysine biosynthesis  *Wbm*01100 Metabolic pathways  *Wbm*01110 Biosynthesis of secondary metabolites  *Wbm*01120 Microbial metabolism in diverse environments  *Wbm*01210 2-Oxocarboxylic acid metabolism  *Wbm*01230 Biosynthesis of amino acids |
|  | Q5GTA6 | *Wbm*0177 | Cytoplasm | 264 | 4-hydroxy-tetrahydrodipicolinate reductase [EC:[1.17.1.8](http://www.genome.jp/dbget-bin/www_bget?ec:1.17.1.8)] | *Wbm*00300 Lysine biosynthesis  *Wbm*01100 Metabolic pathways  *Wbm*01110 Biosynthesis of secondary metabolites  *Wbm*01120 Microbial metabolism in diverse environments  *Wbm*01230 Biosynthesis of amino acids |
|  | Q5GSI7 | *Wbm*0449 | Cytoplasm | 273 | 2,3,4,5-tetrahydropyridine-2-carboxylate N-succinyltransferase [EC:[2.3.1.117](http://www.genome.jp/dbget-bin/www_bget?ec:2.3.1.117)] | *Wbm*00300 Lysine biosynthesis  *Wbm*01100 Metabolic pathways  *Wbm*01120 Microbial metabolism in diverse environments  *Wbm*01230 Biosynthesis of amino acids |
|  | Q5GSB8 | *Wbm*0518 | Cytoplasm | 259 | Diaminopimelate epimerase [EC:[5.1.1.7](http://www.genome.jp/dbget-bin/www_bget?ec:5.1.1.7)] | *Wbm*00300 Lysine biosynthesis  *Wbm*01100 Metabolic pathways  *Wbm*01110 Biosynthesis of secondary metabolites  *Wbm*01120 Microbial metabolism in diverse environments  *Wbm*01230 Biosynthesis of amino acids |
|  | Q5GS80 | *Wbm*0556 | Cytoplasm | 305 | Glutathione synthase [EC:[6.3.2.3](http://www.genome.jp/dbget-bin/www_bget?ec:6.3.2.3)] | *Wbm*00480 Glutathione metabolism  *Wbm*01100 Metabolic pathways |
|  | Q5GTQ6 | *Wbm*0026 | Cytoplasm | 378 | Diaminohydroxyphosphoribosylaminopyrimidine deaminase / 5-amino-6-(5-phosphoribosylamino)uracil reductase [EC:[3.5.4.26](http://www.genome.jp/dbget-bin/www_bget?ec:3.5.4.26) [1.1.1.193](http://www.genome.jp/dbget-bin/www_bget?ec:1.1.1.193)] | *Wbm*00740 Riboflavin metabolism  *Wbm*01100 Metabolic pathways |
|  | Q5GT94 | *Wbm*0189 | Cytoplasm | 142 | 6,7-dimethyl-8-ribityllumazine synthase [EC:[2.5.1.78](http://www.genome.jp/dbget-bin/www_bget?ec:2.5.1.78)] | *Wbm*00740 Riboflavin metabolism  *Wbm*01100 Metabolic pathways |
|  | Q5GTJ9 | *Wbm*0083 | Cytoplasm | 198 | Riboflavin synthase [EC:[2.5.1.9](http://www.genome.jp/dbget-bin/www_bget?ec:2.5.1.9)] | *Wbm*00740 Riboflavin metabolism  *Wbm*01100 Metabolic pathways |
|  | Q5GSN6 | *Wbm*0400 | Cytoplasm | 264 | Inorganic polyphosphate/ATP-NAD kinase (EC:2.7.1.23) | *Wbm*00760 Nicotinate and nicotinamide metabolism  *Wbm*01100 Metabolic pathways |
|  | Q5GTK4 | *Wbm*0078 | Cytoplasm | 125 | Holo-[acyl-carrier protein] synthase [EC:[2.7.8.7](http://www.genome.jp/dbget-bin/www_bget?ec:2.7.8.7)] | *Wbm*00770 Pantothenate and CoA biosynthesis |
|  | Q5GRQ8 | *Wbm*0728 | Cytoplasm | 235 | Uroporphyrinogen-III synthase [EC:[4.2.1.75](http://www.genome.jp/dbget-bin/www_bget?ec:4.2.1.75)] | *Wbm*00860 Porphyrin and chlorophyll metabolism  *Wbm*01100 Metabolic pathways  *Wbm*01110 Biosynthesis of secondary metabolites |
|  | Q5GRR7 | *Wbm*0719 | Cytoplasm | 340 | Ferrochelatase [EC:[4.99.1.1](http://www.genome.jp/dbget-bin/www_bget?ec:4.99.1.1)] | *Wbm*00860 Porphyrin and chlorophyll metabolism  *Wbm*01100 Metabolic pathways  *Wbm*01110 Biosynthesis of secondary metabolites |
|  | Q5GTL0 | *Wbm*0072 | Cytoplasm | 187 | 3-octaprenyl-4-hydroxybenzoate carboxy-lyase UbiX [EC:4.1.1.-] | *Wbm*00130 Ubiquinone and other terpenoid-quinone biosynthesis  *Wbm*01100 Metabolic pathways  *Wbm*01110 Biosynthesis of secondary metabolites |
|  | Q5GTA4 | *Wbm*0179 | Cytoplasm | 389 | 1-deoxy-D-xylulose-5-phosphate reductoisomerase [EC:[1.1.1.267](http://www.genome.jp/dbget-bin/www_bget?ec:1.1.1.267)] | *Wbm*00900 Terpenoid backbone biosynthesis  *Wbm*01100 Metabolic pathways  *Wbm*01110 Biosynthesis of secondary metabolites |
|  | Q5GTB0 | *Wbm*0173 | Cytoplasm | 288 | 4-diphosphocytidyl-2-C-methyl-D-erythritol kinase [EC:[2.7.1.148](http://www.genome.jp/dbget-bin/www_bget?ec:2.7.1.148)] | *Wbm*00900 Terpenoid backbone biosynthesis  *Wbm*01100 Metabolic pathways  *Wbm*01110 Biosynthesis of secondary metabolites |
|  | Q5GRK4 | *Wbm*0782 | Cytoplasm | 429 | E)-4-hydroxy-3-methylbut-2-enyl-diphosphate synthase [EC:[1.17.7.1](http://www.genome.jp/dbget-bin/www_bget?ec:1.17.7.1)] | *Wbm*00900 Terpenoid backbone biosynthesis  *Wbm*01100 Metabolic pathways  *Wbm*01110 Biosynthesis of secondary metabolites |
|  | Q5GSP9 | *Wbm*0387 | Cytoplasm | 142 | DNA-directed RNA polymerase subunit omega [EC:[2.7.7.6](http://www.genome.jp/dbget-bin/www_bget?ec:2.7.7.6)] | *Wbm*00230 Purine metabolism  *Wbm*00240 Pyrimidine metabolism  *Wbm*01100 Metabolic pathways  *Wbm*03020 RNA polymerase |
|  | Q5GSU3 | *Wbm*0342 | Cytoplasm | 121 | Small subunit ribosomal protein S10 | *Wbm*03010 Ribosome |
|  | Q5GSV0 | *Wbm*0335 | Cytoplasm | 206 | Small subunit ribosomal protein S3 | *Wbm*03010 Ribosome |
|  | Q5GSV8 | *Wbm*0327 | Cytoplasm | 131 | Small subunit ribosomal protein S8 | *Wbm*03010 Ribosome |
|  | Q5GSV9 | *Wbm*0326 | Cytoplasm | 181 | Large subunit ribosomal protein L6 | *Wbm*03010 Ribosome |
|  | Q5GSW0 | *Wbm*0325 | Cytoplasm | 123 | Large subunit ribosomal protein L18 | *Wbm*03010 Ribosome |
|  | Q5GRY7 | *Wbm*0649 | Cytoplasm | 172 | Large subunit ribosomal protein L10 | *Wbm*03010 Ribosome |
|  | Q5GTR2 | *Wbm*0020 | Cytoplasm | 121 | Small subunit ribosomal protein S15 | *Wbm*03010 Ribosome |
|  | Q5GSD4 | *Wbm*0502 | Cytoplasm | 186 | Large subunit ribosomal protein L9 | *Wbm*03010 Ribosome |
|  | Q5GSD6 | *Wbm*0500 | Cytoplasm | 260 | Small subunit ribosomal protein S6 | *Wbm*03010 Ribosome |
|  | Q5GTI0 | *Wbm*0102 | Cytoplasm | 117 | Aspartyl-tRNA(Asn)/glutamyl-tRNA(Gln) Amidotransferase subunit C [EC:[6.3.5.6](http://www.genome.jp/dbget-bin/www_bget?ec:6.3.5.6) [6.3.5.7](http://www.genome.jp/dbget-bin/www_bget?ec:6.3.5.7)] | *Wbm*00970 Aminoacyl-tRNA biosynthesis  *Wbm*01100 Metabolic pathways |
|  | Q5GSR6 | *Wbm*0370 | Cytoplasm | 279 | Glycyl-tRNA synthetase alpha chain [EC:[6.1.1.14](http://www.genome.jp/dbget-bin/www_bget?ec:6.1.1.14)] | *Wbm*00970 Aminoacyl-tRNA biosynthesis |
|  | Q5GRI7 | *Wbm*0799 | Cytoplasm | 479 | Lysyl-tRNA synthetase, class I [EC:[6.1.1.6](http://www.genome.jp/dbget-bin/www_bget?ec:6.1.1.6)] | *Wbm*00970 Aminoacyl-tRNA biosynthesis |
|  | Q5GRM6 | *Wbm*0760 | Inner-Membrane | 205 | Signal peptidase I [EC:[3.4.21.89](http://www.genome.jp/dbget-bin/www_bget?ec:3.4.21.89)] | *Wbm*03060 Protein export |
|  | Q5GSE0 | *Wbm*0496 | Cytoplasm | 590 | Ribonuclease E [EC:[3.1.26.12](http://www.genome.jp/dbget-bin/www_bget?ec:3.1.26.12)] | *Wbm*03018 RNA degradation |
|  | Q5GS93 | *Wbm*0543 | Cytoplasm | 231 | DNA polymerase III subunit epsilon [EC:[2.7.7.7](http://www.genome.jp/dbget-bin/www_bget?ec:2.7.7.7)] | *Wbm*00230 Purine metabolism  *Wbm*00240 Pyrimidine metabolism  *Wbm*01100 Metabolic pathways  *Wbm*03030 DNA replication  *Wbm*03430 Mismatch repair  *Wbm*03440 Homologous recombination |
|  | Q5GSD7 | *Wbm*0499 | Cytoplasm | 975 | DNA polymerase III subunit alpha [EC:[2.7.7.7](http://www.genome.jp/dbget-bin/www_bget?ec:2.7.7.7)] | *Wbm*00230 Purine metabolism  *Wbm*00240 Pyrimidine metabolism  *Wbm*01100 Metabolic pathways  *Wbm*03030 DNA replication  *Wbm*03430 Mismatch repair  *Wbm*03440 Homologous recombination |
|  | Q5GSK2 | *Wbm*0434 | Cytoplasm | 494 | DNA polymerase III subunit gamma/tau [EC:[2.7.7.7](http://www.genome.jp/dbget-bin/www_bget?ec:2.7.7.7)] | *Wbm*00230 Purine metabolism  *Wbm*00240 Pyrimidine metabolism  *Wbm*01100 Metabolic pathways  *Wbm*03030 DNA replication  *Wbm*03430 Mismatch repair  *Wbm*03440 Homologous recombination |
|  | Q5GSK7 | *Wbm*0429 | Cytoplasm | 393 | DNA polymerase III subunit beta [EC:[2.7.7.7](http://www.genome.jp/dbget-bin/www_bget?ec:2.7.7.7)] | *Wbm*00230 Purine metabolism  *Wbm*00240 Pyrimidine metabolism  *Wbm*01100 Metabolic pathways  *Wbm*03030 DNA replication  *Wbm*03430 Mismatch repair  *Wbm*03440 Homologous recombination |
|  | Q5GSY6 | *Wbm*0299 | Nucleoid | 483 | Replicative DNA helicase [EC:[3.6.4.12](http://www.genome.jp/dbget-bin/www_bget?ec:3.6.4.12)] | *Wbm*03030 DNA replication |
|  | Q5GT89 | *Wbm*0194 | Cytoplasm | 586 | DNA primase [EC:2.7.7.-] | *Wbm*03030 DNA replication |
|  | Q5GS47 | *Wbm*0589 | Cytoplasm | 271 | Formamidopyrimidine-DNA glycosylase [EC:[3.2.2.23](http://www.genome.jp/dbget-bin/www_bget?ec:3.2.2.23)[4.2.99.18](http://www.genome.jp/dbget-bin/www_bget?ec:4.2.99.18)] | *Wbm*03410 Base excision repair |
|  | Q5GTF9 | *Wbm*0124 | Cytoplasm | 579 | Single-stranded-DNA-specific exonuclease [EC:3.1.-.-] | *Wbm*03410 Base excision repair  *Wbm*03430 Mismatch repair  *Wbm*03440 Homologous recombination |
|  | Q5GSR4 | *Wbm*0372 | Cytoplasm | 606 | Excinuclease ABC subunit C | *Wbm*03420 Nucleotide excision repair |
|  | Q5GS08 | *Wbm*0628 | Cytoplasm | 638 | DNA helicase II / ATP-dependent DNA helicase PcrA [EC:[3.6.4.12](http://www.genome.jp/dbget-bin/www_bget?ec:3.6.4.12)] | *Wbm*03420 Nucleotide excision repair  *Wbm*03430 Mismatch repair |
|  | Q5GT34 | *Wbm*0251 | Cytoplasm | 196 | Holliday junction DNA helicase RuvA [EC:[3.6.4.12](http://www.genome.jp/dbget-bin/www_bget?ec:3.6.4.12)] | *Wbm*03440 Homologous recombination |
|  | Q5GRT9 | *Wbm*0697 | Cytoplasm | 162 | Crossover junction endodeoxyribonuclease RuvC [EC:[3.1.22.4](http://www.genome.jp/dbget-bin/www_bget?ec:3.1.22.4)] | *Wbm*03440 Homologous recombination |
|  | Q5GRP1 | *Wbm*0745 | Cytoplasm | 786 | Primosomal protein N' (replication factor Y) (superfamily II helicase) [EC:3.6.4.-] | *Wbm*03440 Homologous recombination *Wbm*03440 Homologous recombination |
|  | Q5GS66 | *Wbm*0570 | Cytoplasm | 339 | D-alanine-D-alanine ligase [EC:[6.3.2.4](http://www.genome.jp/dbget-bin/www_bget?ec:6.3.2.4)] | *Wbm*00550 Peptidoglycan biosynthesis  *Wbm*01100 Metabolic pathways |
|  | Q5GS79 | *Wbm*0557 | Cytoplasm | 343 | UDP-N-acetylglucosamine--N-acetylmuramyl-(pentapeptide) pyrophosphoryl-undecaprenol N-acetylglucosamine transferase [EC:[2.4.1.227](http://www.genome.jp/dbget-bin/www_bget?ec:2.4.1.227)] | *Wbm*00550 Peptidoglycan biosynthesis  *Wbm*01100 Metabolic pathways |
|  | Q5GTK7 | *Wbm*0075 | Inner-membrane | 521 | Penicillin-binding protein 2 | *Wbm*00312 Beta-Lactam resistance  *Wbm*00550 Peptidoglycan biosynthesis |
|  | Q5GT97 | *Wbm*0186 | Inner-membrane | 293 | Preprotein translocase subunit SecF | *Wbm*03060 Protein export  *Wbm*03070 Bacterial secretion system |
|  | Q5GSW3 | *Wbm*0322 | Inner-membrane | 442 | Preprotein translocase subunit SecY | *Wbm*03060 Protein export  *Wbm*03070 Bacterial secretion system |
|  | Q5GT61 | *Wbm*0222 | Cytoplasm | 144 | Preprotein translocase subunit YajC | *Wbm*03060 Protein export  *Wbm*03070 Bacterial secretion system |
|  | Q5GSY8 | *Wbm*0297 | Inner-membrane | 255 | Sec-independent protein translocase protein TatC | *Wbm*03060 Protein export  *Wbm*03070 Bacterial secretion system |
|  | Q5GTF5 | *Wbm*0128 | Inner-membrane | 475 | Two-component system, cell cycle sensor histidine kinase PleC [EC:[2.7.13.3](http://www.genome.jp/dbget-bin/www_bget?ec:2.7.13.3)] | *Wbm*02020 Two-component system |
|  | Q5GS11 | *Wbm*0625 | Inner-membrane | 177 | NADH-quinone oxidoreductase subunit J [EC:[1.6.5.3](http://www.genome.jp/dbget-bin/www_bget?ec:1.6.5.3)] | *Wbm*00190 Oxidative phosphorylation |
|  | Q5GS12 | *Wbm*0624 | Inner-membrane | 102 | NADH-quinone oxidoreductase subunit K [EC:[1.6.5.3](http://www.genome.jp/dbget-bin/www_bget?ec:1.6.5.3)] | *Wbm*00190 Oxidative phosphorylation |
|  | Q5GS13 | *Wbm*0623 | Inner-membrane | 618 | NADH-quinone oxidoreductase subunit L [EC:[1.6.5.3](http://www.genome.jp/dbget-bin/www_bget?ec:1.6.5.3)] | *Wbm*00190 Oxidative phosphorylation |
|  | Q5GSF0 | *Wbm*0486 | Inner-membrane | 125 | Succinate dehydrogenase cytochrome b556 subunit | *Wbm*00020 Citrate cycle (TCA cycle)  *Wbm*00190 Oxidative phosphorylation  *Wbm*01100 Metabolic pathways  *Wbm*01110 Biosynthesis of secondary metabolites  *Wbm*01120 Microbial metabolism in diverse environments  *Wbm*01200 Carbon metabolism |
|  | Q5GSF1 | *Wbm*0485 | Inner-membrane | 121 | Succinate dehydrogenase membrane anchor subunit | *Wbm*00020 Citrate cycle (TCA cycle)  *Wbm*00190 Oxidative phosphorylation  *Wbm*01100 Metabolic pathways  *Wbm*01110 Biosynthesis of secondary metabolites  *Wbm*01120 Microbial metabolism in diverse environments  *Wbm*01200 Carbon metabolism |
|  | Q5GS22 | *Wbm*0614 | Cytoplasm | 326 | 3-oxoacyl-[acyl-carrier-protein] synthase III [EC:[2.3.1.180](http://www.genome.jp/dbget-bin/www_bget?ec:2.3.1.180)] | *Wbm*00061 Fatty acid biosynthesis  *Wbm*01100 Metabolic pathways  *Wbm*01212 Fatty acid metabolism |
|  | Q5GSL3 | *Wbm*0423 | Inner-membrane | 191 | Glycerol-3-phosphate acyltransferase PlsY [EC:[2.3.1.15](http://www.genome.jp/dbget-bin/www_bget?ec:2.3.1.15)] | *Wbm*00561 Glycerolipid metabolism  *Wbm*00564 Glycerophospholipid metabolism  *Wbm*01100 Metabolic pathways |
|  | Q5GSL1 | *Wbm*0425 | Inner-membrane | 256 | CDP-diacylglycerol---serine O-phosphatidyltransferase [EC:[2.7.8.8](http://www.genome.jp/dbget-bin/www_bget?ec:2.7.8.8)] | *Wbm*00260 Glycine, serine and threonine metabolism  *Wbm*00564 Glycerophospholipid metabolism  *Wbm*01100 Metabolic pathways |
|  | Q5GSL0 | *Wbm*0426 | Cytoplasm | 230 | Phosphatidylserine decarboxylase [EC:[4.1.1.65](http://www.genome.jp/dbget-bin/www_bget?ec:4.1.1.65)] | *Wbm*00564 Glycerophospholipid metabolism |
|  | Q5GS70 | *Wbm*0566 | Cytoplasm | 318 | Thioredoxin reductase (NADPH) [EC:[1.8.1.9](http://www.genome.jp/dbget-bin/www_bget?ec:1.8.1.9)] | *Wbm*00240 Pyrimidine metabolism  *Wbm*00450 Selenocompound metabolism |
|  | Q5GSL9 | *Wbm*0417 | Inner-Membrane | 154 | Signal peptidase II [EC:[3.4.23.36](http://www.genome.jp/dbget-bin/www_bget?ec:3.4.23.36)] | *Wbm*03060 Protein export |
|  | Q5GSX3 | *Wbm*0312 | Periplasm | 217 | 3,4-dihydroxy 2-butanone 4-phosphate synthase [EC:[4.1.99.12](http://www.genome.jp/dbget-bin/www_bget?ec:4.1.99.12)] | *Wbm*00740 Riboflavin metabolism  *Wbm*01100 Metabolic pathways |
|  | Q5GTN6 | *Wbm*0046 | Periplasm | 309 | 4-hydroxy-3-methylbut-2-enyl diphosphate reductase (EC:1.17.1.2) | *Wbm*00900 Terpenoid backbone biosynthesis  *Wbm*01100 Metabolic pathways  *Wbm*01110 Biosynthesis of secondary metabolites |
|  | Q5GS61 | *Wbm*0575 | Periplasm | 411 | Outer membrane channel protein | *Wbm*00312 beta-Lactam resistance  *Wbm*03070 Bacterial secretion system |

**Supplementary Table 4:** Druggable therapeutic targets of *wBm.* Druggability was analyzes against the DrugBank database and TTD database result.

| **Sl. No.** | **Protein ID** | **All Carrier proteins** | **All Drugbank enzymes** | **All DrugBank target** | **TDR** |
| --- | --- | --- | --- | --- | --- |
|  | Q5GS61 | P15090 Fatty acid-binding protein, adipocyte (DB02153; B02776; DB03009; DB03766) |  | P02930 Outer membrane protein TolC (DB03350) |  |
|  | Q5GTP1 |  | CAD protein (DB00130) | drugbank|P33221 Phosphoribosylglycinamide formyltransferase 2 (DB02236; DB02930; DB03434; DB03909; DB04395) | Q92210|PUR6_CANAL Phosphoribosylaminoimidazole carboxylase OS=Candida albicans  (strain SC5314 / ATCC MYA-2876) GN=ADE2 PE=3 SV=2 |
|  | Q5GRP6 |  |  | Q9S400 3-phosphoshikimate 1-carboxyvinyltransferase (DB04328; DB04539) | P0A751|MURA_SHIFL UDP-N-acetylglucosamine 1-carboxyvinyltransferase OS=Shigella  flexneri GN=murA PE=3 SV=1 |
|  | Q5GRK8 |  |  | P61432 UDP-N-acetylenolpyruvoylglucosamine reductase (DB03147) | MURB_STAAU UDP-N-acetylenolpyruvoylglucosamine reductase OS=Staphylococcus  aureus GN=murB PE=1 SV=1 |
|  | Q5GSC8 |  |  | Q8DNV6 UDP-N-acetylmuramoyl-tripeptide--D-alanyl-D-alanine ligase (DB06970) |  |
|  | Q5GS66 |  |  | Q5HEB7 D-alanine--D-alanine ligase (DB07805) | DDL_MYCTU D-alanine--D-alanine ligase OS=Mycobacterium tuberculosis (strain  ATCC 25618 / H37Rv) GN=ddl PE=1 SV=1 |
|  | Q5GT47 |  |  | Q8DNV6 UDP-N-acetylmuramoyl-tripeptide--D-alanyl-D-alanine ligase (DB06970) |  |
|  | Q5GTK7 |  |  | P0AD65 Penicillin-binding protein 2 (DB00303; DB00438; DB00948; DB01163;  DB01327; DB01328; DB01329; DB01413; DB01415; DB01598) | PBP2_ECOLI Penicillin-binding protein 2 OS=Escherichia coli (strain K12)  GN=mrdA PE=3 SV= |
|  | Q5GSZ5 |  |  | P08506 D-alanyl-D-alanine carboxypeptidase DacC (DB00274; DB00303; DB00430;  DB01329; DB01331) |  |
|  | Q5GT03 |  |  | Q7BK04 Cag alpha (DB02930) |  |
|  | Q5GT10 |  |  | P0A1P6 Glutamine synthetase (DB02663) |  |
|  | Q5GTF5 |  |  | PPH (DB04066) | O74271_CANAX Histidine kinase OS=Candida albicans GN=chik1 PE=4 SV=1 |
|  | Q5GT99 |  |  | O32393 Adenylate cyclase (DB02355; DB02596; DB07706) | O74271_CANAX Histidine kinase OS=Candida albicans GN=chik1 PE=4 SV=1 |
|  | Q5GRI5 |  |  | Phosphopantetheine adenylyltransferase (DB01992; DB03170; DB03912)  15 | COAD_SHIFL Phosphopantetheine adenylyltransferase OS=Shigella flexneri GN=coaD  PE=3 SV=1 |
|  | Q5GTH4 |  |  | Q511225 Outer membrane pro617tein p64k or PM-6 (DB03147) |  |
|  | Q5GTJ4 |  |  | P43889 Bifunctional protein GlmU (DB08344) |  |
|  | Q5GSI1 |  |  | P24182 Biotin carboxylase (DB08074; DB08075; DB08076; DB08144; DB08145;  DB08146; DB08318) | ACACA_HUMAN Acetyl-CoA carboxylase 1 OS=Homo sapiens GN=ACACA PE=1 SV=2 |
|  | Q5GSM8 |  |  | P00943 Triosephosphate isomerase (DB02726) | TPIS_SHIFL Triosephosphate isomerase OS=Shigella flexneri GN=tpiA PE=3 SV=1 |
|  | Q5GSF0 |  |  | P69054 Succinate dehydrogenase cytochrome b556 subunit (DB04631; DB07671;  DB08690) |  |
|  | Q5GSX1 |  |  | P25705 ATP synthase subunit alpha, mitochondrial (DB04216; DB07384;  DB07394; DB08399; DB08629) | VATA_HUMAN V-type proton ATPase catalytic subunit A OS=Homo sapiens GN=ATP6V1A |
|  | Q5GS22 |  |  | Q820T1 3-oxoacyl-[acyl-carrier-protein] synthase 3 (DB07429) | FABH_ECOLI 3-oxoacyl-[acyl-carrier-protein] synthase 3 OS=Escherichia coli  (strain K12) GN=fabH PE=1 SV=1 |
|  | Q5GRP7 |  |  | Q9ZGI2 Type I polyketide synthase PikAIV (DB07703; DB08431; DB08759) | FABF_SHIFL 3-oxoacyl-[acyl-carrier-protein] synthase 2 OS=Shigella flexneri  GN=fabF PE=3 SV=2 |
|  | Q5GTN0 |  |  | O25928 3-hydroxyacyl-[acyl-carrier-protein] dehydratase FabZ (DB07445) |  |
|  | Q5GRN3 |  |  | Q9FBC5 Trans-2-enoyl-ACP reductase II (DB08657) |  |
|  | Q5GSN9 |  |  | Q2QJL3 N5-carboxyaminoimidazole ribonucleotide mutase (DB04272) | PUR6_CANAL Phosphoribosylaminoimidazole carboxylase OS=Candida albicans  (strain SC5314 / ATCC MYA-2876) GN=ADE2 PE=3 SV=2 |
|  | Q5GSW7 |  |  | Q5SHR6 DNA-directed RNA polymerase subunit alpha (DB08266) |  |
|  | Q5GRJ9 |  |  | P25971 Orotidine 5'-phosphate decarboxylase (DB03685) |  |
|  | Q5GTA6 |  |  | P04036 4-hydroxy-tetrahydrodipicolinate reductase (DB03969; DB04267) |  |
|  | Q5GSI7 |  |  | Bifunctional protein GlmU (DB08344) |  |
|  | Q5GS70 |  |  | Thioredoxin reductase (DB03147) | TRXB_MYCLE Bifunctional thioredoxin reductase/thioredoxin OS=Mycobacterium  leprae (strain TN) GN=trxB/A PE=3 SV=1 |
|  | Q5GT94 |  |  | P11998 6,7-dimethyl-8-ribityllumazine synthase (DB04162) |  |
|  | Q5GTJ9 |  |  | P0AFU8 Riboflavin synthase (DB00140) |  |
|  | Q5GTK4 |  |  | P96618 Holo-[acyl-carrier-protein] synthase (DB01992; DB04447) |  |
|  | Q5GRR7 |  |  | Q7KZA3 Ferrochelatase (DB02659) |  |
|  | Q5GTL0 |  |  | P69772 Probable aromatic acid decarboxylase (DB03247) |  |
|  | Q5GTN6 |  |  | P62623 4-hydroxy-3-methylbut-2-enyl diphosphate reductase (DB01785; DB04714) |  |
|  | Q5GSU3 |  |  | Q5SHN7 30S ribosomal protein S10 (DB01421; DB08185) |  |
|  | Q5GSV0 |  |  | P80372 30S ribosomal protein S3 (DB08185) |  |
|  | Q5GSV8 |  |  | Q5SHQ2 30S ribosomal protein S8 (DB08185) |  |
|  | Q5GS93 |  |  | P03007 DNA polymerase III subunit epsilon (DB01643) |  |
|  | Q5GSK2 |  |  | P06710 DNA polymerase III subunit tau (DB02930) |  |
|  | Q5GSK7 |  |  | P0A988 DNA polymerase III subunit beta (DB06998) |  |
|  | Q5GSY6 |  |  | P03692 DNA primase/helicase (DB02452; DB03222) | Q6GKS9_STAAR DnaB-like helicase OS=Staphylococcus aureus (strain MRSA252)  GN=dnaC PE=4 SV=1 |
|  | Q5GSN3 |  |  | Q9X519 2,3-bisphosphoglycerate-independent phosphoglycerate mutase (DB01709; DB04510) | GPMI_MYCGE 2,3-bisphosphoglycerate-independent phosphoglycerate mutase OS=Mycoplasma  genitalium (strain ATCC 33530 / G-37 / NCTC 10195) |
|  | Q5GTG5 |  |  | P45066 UDP-N-acetylmuramate--L-alanine ligase (DB01673; DB03909;DB04395) |  |
|  | Q5GSE4 |  |  | P22188 UDP-N-acetylmuramoyl-L-alanyl-D-glutamate--2,6-diaminopimelate ligase (DB02314; DB03590; DB03801) |  |
|  | Q5GS79 |  |  | P17443 UDP-N-acetylglucosamine--N-acetylmuramyl-(pentapeptide) pyrophosphoryl-undecaprenol  N-acetylglucosamine transferase (DB02196) | MURG_ECOLI UDP-N-acetylglucosamine--N-acetylmuramyl-(pentapeptide) pyrophosphoryl-undecaprenol  N-acetylglucosamine transferase OS=Escherichia |
|  | Q5GS12 |  |  | P03901 NADH-ubiquinone oxidoreductase chain 4L (DB00157) |  |
|  | Q5GS13 |  |  | P03915 NADH-ubiquinone oxidoreductase chain 5 (DB00157; DB05382) |  |
|  | Q5GTP0 |  |  | P44801 Aspartate-semialdehyde dehydrogenase (DB03461; DB03502; DB04498) | DHAS2_VIBCH Aspartate-semialdehyde dehydrogenase 2 OS=Vibrio cholerae serotype  O1 (strain ATCC 39315 / El Tor Inaba N16961) GN=asd2 PE=1 |
|  | Q5GTQ6 |  |  | P16006 Deoxycytidylate deaminase (DB04280) |  |
|  | Q5GTA4 |  |  | P45568 1-deoxy-D-xylulose 5-phosphate reductoisomerase (DB02496; DB02948; DB03649; DB04272) |  |
|  | Q5GTB0 |  |  | Q83LD8 4-diphosphocytidyl-2-C-methyl-D-erythritol kinase (DB03687; DB04395) |  |
|  | Q5GRY7 |  |  | P0A7J6 50S ribosomal protein L10 (DB00778; DB01190; DB01211; DB01369; DB01627) |  |
|  | Q5GRM6 |  |  | P00803 Signal peptidase I (DB01934; DB02080; DB06904) | LEP_ECOLI Signal peptidase I OS=Escherichia coli (strain K12) GN=lepB PE=1  SV=2 |
|  | Q5GTF9 |  |  | P22256 4-aminobutyrate aminotransferase GabT (DB02142; DB02783) |  |
|  | Q5GTA1 |  |  |  | Q9NGT0_PLAFA Glutamate dehydrogenase OS=Plasmodium falciparum GN=gdh PE=4 |
|  | Q5GSL1 |  |  |  | PSS_MYCTO CDP-diacylglycerol--serine O-phosphatidyltransferase OS=Mycobacterium  tuberculosis (strain CDC 1551 / Oshkosh) GN=pssA PE=3 |
|  | Q5GS87 |  |  |  | P0A840|SURE_ECOLI 5'/3'-nucleotidase SurE OS=Escherichia coli (strain K12) GN=surE  PE=1 SV=1 |
|  | Q5GSX3 |  |  |  | RIBB_SHIFL 3,4-dihydroxy-2-butanone 4-phosphate synthase OS=Shigella flexneri  GN=ribB PE=3 SV=1 |
|  | Q5GRI7 |  |  |  | SYK_RICPR Lysine--tRNA ligase OS=Rickettsia prowazekii (strain Madrid E) |
